# Comparison of US patient, rheumatologist, and dermatologist perceptions of psoriatic disease symptoms: results from the DISCONNECT study

**DOI:** 10.1186/s13075-018-1601-4

**Published:** 2018-05-31

**Authors:** M. Elaine Husni, Anthony Fernandez, Brett Hauber, Rakesh Singh, Joshua Posner, Jessie Sutphin, Arijit Ganguli

**Affiliations:** 1 Rheumatology Department, Cleveland Clinic Foundation, Cleveland, OH USA; 2 Dermatology and Pathology Department, Cleveland Clinic Foundation, Cleveland, OH USA; 3 RTI Health Solutions, 3040 Cornwallis Road, Post Office Box 12194, Research Triangle Park, NC 27709-2194 USA; 4 AbbVie, North Chicago, IL USA; 5 Yale School of Management, New Haven, CT USA

**Keywords:** Psoriatic disease, Psoriasis, Psoriatic arthritis, Skin symptoms, Joint symptoms, Patients, Physicians

## Abstract

**Background:**

The perceived bother of skin and joint-related manifestations of psoriatic disease may differ among patients, rheumatologists, and dermatologists. This study identified and compared the patient and dermatologist/rheumatologist-perceived bother of psoriatic disease manifestations.

**Methods:**

Online surveys were administered to patients with both psoriasis and psoriatic arthritis and to dermatologists and rheumatologists. Object-case best–worst scaling was used to identify the most and least bothersome items from a set of five items in a series of questions. Each item set was drawn from 20 items describing psoriatic disease skin and joint symptoms and impacts on daily activities. Survey responses were analyzed using random-parameters logit models for each surveyed group, yielding a relative-bother weight (RBW) for each item compared with joint pain, soreness, or tenderness.

**Results:**

Surveys were completed by 200 patients, 150 dermatologists, and 150 rheumatologists. Patients and physicians agreed that joint pain, soreness, and tenderness are among the most bothersome manifestations of psoriatic disease (RBW 1.00). For patients, painful, inflamed, or broken skin (RBW 1.03) was more bothersome, while both rheumatologists and dermatologists considered painful skin much less bothersome (RBW 0.17 and 0.22, respectively) than joint pain. Relative to joint pain, rheumatologists were more likely to perceive other joint symptoms as bothersome, while dermatologists were more likely to perceive other skin symptoms as bothersome.

**Conclusions:**

This study has identified important areas of discordance both between patients and physicians and between rheumatologists and dermatologists about the relative bother of a comprehensive set of psoriatic disease symptoms and functional impacts. Both physician specialists should ask patients which manifestations of psoriatic disease are most bothersome to them, as these discussions may have important implications for drug and other patient management options.

**Electronic supplementary material:**

The online version of this article (10.1186/s13075-018-1601-4) contains supplementary material, which is available to authorized users.

## Significance and innovations

- This study determines the relative bothersomeness of a wide range of symptoms and activity limitations associated with psoriatic disease from the perspectives of patients, dermatologists, and rheumatologists in the US.
- The perceived bother of many of the manifestations of psoriatic disease may differ among patients, rheumatologists, and dermatologists.
- Patients and physicians agree that joint pain, soreness, and tenderness are among the most bothersome manifestations of psoriatic disease. Patients consider painful, inflamed, or broken skin to be more bothersome than joint pain, soreness, or tenderness, whereas both dermatologists and rheumatologists consider painful, inflamed, or broken skin much less bothersome than joint pain, soreness, or tenderness.
- There are also differences between rheumatologists and dermatologists in their perceptions of the bother to patients of different psoriatic disease manifestations, with rheumatologists more concerned about joint-related symptoms and dermatologists more concerned about skin-related symptoms.
- Rheumatologists and dermatologists should discuss the bother of all manifestations of psoriatic disease with their patients to ensure optimal care.

## Background

The skin and joints are the two most common organs involved with psoriatic disease, which includes psoriasis and psoriatic arthritis. Psoriasis most commonly presents as well-demarcated erythematous plaques with silvery-white scales that produce plaque-related pain, changes in skin appearance, and pruritus [1]. Psoriatic arthritis is an inflammatory form of arthritis with symptoms including swelling, stiffness, and pain in the affected joint [2, 3]. Both psoriatic arthritis and psoriasis originate from an abnormal response in the immune system; thus, treatment is focused on modulating the immune response. Although concurrent treatment of psoriatic arthritis and psoriasis may be convenient if it allows patients to use a single agent [4], choice of therapy for those with psoriasis and psoriatic arthritis should address as many manifestations of psoriatic disease as possible [5].

Psoriatic arthritis frequently occurs between 8 and 12 years after the onset of psoriasis and is known to affect approximately 30% of those with psoriasis [6]. However, in approximately 15% of cases, psoriatic arthritis and psoriasis occur simultaneously or psoriatic arthritis precedes psoriasis [3]. Although the skin will heal without scarring or other permanent change even when treatment is delayed, patients with psoriatic arthritis can have persistent inflammation, progressive and chronic joint damage, severe physical limitations, disability, and increased mortality [7].

It is possible that patients and their physicians have different perceptions of the bother caused by different symptoms. Recent studies in Europe and Canada comparing psoriatic arthritis patients’ global assessments of their disease activity with physician global assessments have demonstrated this discordance between patients and physicians [8–11]. Patients with psoriatic arthritis and psoriasis may perceive both skin and joint symptoms as equally bothersome and important to manage because of their impact on daily activities [12]. However, it is possible that physicians’ perceptions of the bother of symptoms to patients, and thus physicians’ treatment priorities, are influenced by their specialty. In particular, dermatologists may perceive skin symptoms as being more bothersome to patients than joint symptoms and, therefore, may delay in referring their patients to rheumatologists until the joint symptoms are advanced and more difficult to treat [6, 7].

The objective of this study (the DISCONNECT study) was to compare perceptions of the relative bother of skin and joint symptoms and activity limitations for patients, dermatologists, and rheumatologists in the United States (US). An understanding of any disconnect or discordance in perceptions among patients and physicians by specialty might provide guidance for treatment choice and efficacy assessment, as well as insights into the reasons for the delayed referral from dermatologists to rheumatologists.

## Methods

### Overview

The study was conducted through responses of patients and physicians to a survey including questions designed using a best–worst scaling (BWS) technique. This technique allowed us to estimate the relative importance to patients, dermatologists, and rheumatologists of varying types of skin and joint symptoms and their impacts on patients’ daily activities. Object-case BWS, a rigorous approach grounded in random utility theory, a well-tested theory of human decision-making [13], was used in this study. BWS was used to estimate perception of relative bother to patients of the different manifestations of psoriatic disease as well as the relative bother of these manifestations as perceived by dermatologists and rheumatologists involved in their care. BWS is a method for eliciting stated preferences that asks respondents to indicate which item is “best” and which item is “worst” from among a subset of items of interest in a series of questions [13, 14]. In each BWS question in this study, respondents were asked to choose one item as most bothersome (i.e., “worst”) and one item as least bothersome (i.e., “best”) from different combinations of five of the 20 items included in the study. In object-case BWS, the items included represent a mutually exclusive set of descriptors of the impact of a disease without presenting different levels for each item. Two surveys were created for this study, a patient survey and a physician survey. Both descriptive analyses and multivariate regressions using random-parameters logit (RPL) models were used to derive estimates of relative bother of the different items. Subgroup analyses were also performed for patients subdivided by age, gender, severity of skin or joint symptoms, time since diagnosis, and current treatment.

### Survey instrument

Items for the BWS questions in the surveys were chosen based on: a review of published literature estimating preferences for treatments for psoriatic arthritis and/or psoriasis symptoms that included studies that used items derived from consultation with patients [15–18]; a review of the development and validation of disease-specific quality of life and patient-reported outcome measures (Dermatology Life Quality Index [19] and Psoriatic Arthritis Impact of Disease [20–22]); and consultation with clinical experts. The items in the patient and physician surveys included seven dermatologic symptoms (skin symptoms), seven rheumatologic symptoms (joint symptoms), and six impacts of psoriatic arthritis and psoriasis on daily activities (daily activities) (Table 1). The three categories of manifestations were determined by the study team based on a review of the literature. In addition, the study team attempted to include an approximately equal number of manifestations in each of the three groups. The wording used to describe the items varied slightly between the patient and physician surveys.

**Table 1:** Items included in the patient and physician^a^ surveys

Category	Item	Description
Skin symptoms	Itching skin	Physically irritated skin resulting in the urge to scratch
	Redness of skin	Red or salmon-pink color of psoriasis-affected skin
	Flaking skin	Skin shedding
	Painful skin	Painful, inflamed, or broken skin
	Nail problems	Discoloration or pitting of the fingernails or toenails or separation of the nail from the nail bed
	Difficulty choosing clothing	Skin problems influencing the clothing you wear
	Embarrassment	Being embarrassed or self-conscious because of your skin
Joint symptoms	Joint pain, soreness, or tenderness	Stiffness, pain, throbbing, swelling, and tenderness in one or more joints
	Swelling of fingers or toes	Sausage-like swelling of one or more fingers or toes
	Fatigue	Tiredness and lack of energy that does not go away with sleep
	Morning stiffness	Stiffness after resting that makes it difficult to move your joints
	Eye problems	Eye swelling, redness in or around your eyes, eye pain, and/or blurry vision
	Difficulty dressing	Difficulty tying shoelaces and buttoning your clothes
	Difficulty walking	Difficulty walking at a normal speed
Impacts on daily activities	Difficulty with work or school activities	Difficulty doing your normal work or schoolwork because of your psoriasis or psoriatic arthritis
	Difficulty with social or leisure activities	Difficulty doing your normal social or leisure activities because of your psoriasis or psoriatic arthritis
	Difficulty going shopping or doing housework or yard work	Difficulty going shopping or looking after your home or yard because of your psoriasis or psoriatic arthritis
	Difficulty sleeping	Having poor sleep quality or sleep interruptions because of your psoriasis or psoriatic arthritis
	Discomfort while doing everyday tasks	Discomfort doing everyday tasks, such as eating, bathing, or going to the bathroom, because of your psoriasis or psoriatic arthritis
	Problems with relationships	Problems with partner, close friends, or family because of your psoriasis or psoriatic arthritis

^a^ In the physician survey, the word “your” was removed or substituted with “the patient’s” or “the” as appropriate but it otherwise kept the same wording. See Additional file 2 for the full physician survey

**Fig. 1:**
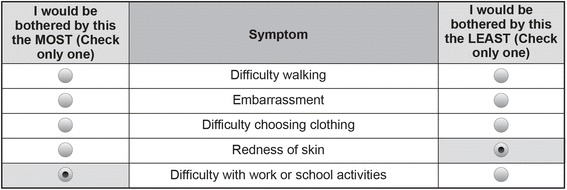
Example of a best–worst scaling question from the patient survey: “Please indicate which one of the 5 symptoms listed below would bother you the most, and which one would bother you the least if you experienced these symptoms”

The patient survey included 16 different BWS questions. In each BWS question, patients were asked to identify the most and least bothersome symptom from a five-item subset of the 20 items if they were to experience all five symptoms. An example of a BWS question presented to patients is shown in Fig. 1. Each physician survey included only eight different BWS questions, but these were repeated for two of three possible patient profiles (Table 2). Physicians were asked to indicate which of a five-item group of the 20 items listed would be most and least bothersome to a patient if the patient experienced all five symptoms. The five items for each BWS question for both the patients and the physicians were determined using an experimental design developed using Sawtooth Software SSI Web version 8.4.4. The order in which each BWS question appeared was randomized to mitigate possible ordering effects.

**Table 2:** Patient profiles in the physician survey

Patient profile	Description
Patient 1 (mild)	■ Each of these patients is [gender] ■ Each of these patients has plaques of mild severity covering 2% BSA ■ Each of these patients has four painful, swollen, or tender joints
Patient 2 (moderate)	■ Each of these patients is [gender] ■ Each of these patients has plaques of moderate severity covering 7% BSA ■ Each of these patients has eight painful, swollen, or tender joints
Patient 3 (severe)	■ Each of these patients is [gender] ■ Each of these patients has plaques of severe severity covering 15% BSA ■ Each of these patients has 11 painful, swollen, or tender joints

*BSA* body surface area

In addition to the BWS questions, surveys included screening questions to ensure eligibility and obtain informed consent. Both surveys also elicited standard demographic information. The patient survey asked about treatments used and whether the respondent had personal experience with the items included in the BWS questions and, if so, asked about item severity and impact. The physician survey elicited information about medical experience related to treating psoriatic arthritis and psoriasis. The complete patient survey instrument is presented in Additional file 1. The complete physician survey instrument is provided in Additional file 2.

### Qualitative pretesting

Qualitative pretesting of the survey instruments was conducted by trained interviewers in face-to-face interviews with a convenience sample of adult patients (*n* = 15), dermatologists (*n* = 8), and rheumatologists (*n* = 7). Pretesting was conducted to ensure that survey items were relevant to respondents, that descriptions of the items were understandable to patients, that no important items were omitted, and that respondents were willing and able to choose the most and least bothersome symptoms. Information gathered during patient and physician pretest interviews was used to refine the final survey instruments, including minor changes in the wording of each item to improve its relevance to patients, dermatologists, and rheumatologists.

### Study sample

The Nielsen Company (US), LLC, a market research company that has several health panels available for survey research in the US in different disease areas, sent targeted email invitations to patients with self-reported psoriasis and/or psoriatic arthritis and to physicians in the US who are members of existing online US health care panels. Patients recruited from these panels were aged 18 years or older with self-reported physician diagnoses of both psoriatic arthritis and psoriasis. Physicians were board-certified or board-eligible rheumatologists or dermatologists and reported spending at least 50% of their time seeing patients.

The pretest interviews and the survey were approved by RTI International’s Office of Research Protection. All respondents in this study were required to provide informed consent at the time of completing the survey.

### Statistical analyses

Responses to all questions in the surveys were summarized using descriptive statistics, with the exception of informed-consent and BWS questions. Skipped questions were coded as missing data points and noted in the summary statistics. BWS data were analyzed using the RPL regression model presented by Yuan et al. [23] based on the assumption that choices recorded from BWS questions reflected two independent decisions for each set of items (“best” and “worst”). To facilitate estimation of a model that considers both decisions, it was assumed that selection of an item as most bothersome and selection of an item as least bothersome reflected equal deviations in importance from the mean importance of the 20 study items, albeit in opposite directions. An RPL model mitigates bias in BWS data from unobserved preference heterogeneity among respondents by estimating a distribution of log-odds importance weights across respondents in the sample and accounting for within-sample correlation when respondents answer multiple questions [24, 25]. Relative-bother weights (RBWs) were calculated from log-odds importance weights using a probability-based rescaling procedure [26]. RBWs were estimated separately for patients, rheumatologists, and dermatologists.

Although an RPL model controls for response heterogeneity across respondents within a sample, it does not explain how response patterns vary systematically with observable respondent characteristics. To help explain response heterogeneity, we estimated separate RPL models for 11 mutually exclusive pairs of patient subgroups and tested for differences in estimated bother weights across each subgroup pair. The subgroups included, among others, age, gender, self-rated severity of skin or joint symptoms, time since diagnosis, and current treatment. Table C-1 in Additional file 3 presents the patient subgroup pairs.

To understand how physicians’ assessments of relative bother varied across patient types, RBWs for each of the three patient types were estimated for both rheumatologists and dermatologists.

## Results

### Respondent characteristics and experience with survey items

Nielsen sent email invitations to a national sample of 16,624 individuals who were members of online health care panels and who had previously indicated that they had psoriasis and/or psoriatic arthritis. A total of 428 individuals accessed the survey screener, 229 (54% of those screened) were eligible, 227 (99%) consented to participate, and 200 (88%) completed the survey. Table 3 presents their characteristics. Approximately 26% of patients were diagnosed with psoriasis less than 1 year ago, 16% were diagnosed 5–10 years ago, and 15% were diagnosed 10 or more years ago. Approximately 37% of patients were diagnosed with psoriatic arthritis less than 1 year ago, 11% were diagnosed 5–10 years ago, and 6% were diagnosed 10 or more years ago. Self-reported mean severity of both skin and joint symptoms in the last week was between 6 and 7 on a scale from 1 to 10, and 39% of patients had used injectable medications and 17% had used infused medications. All patients had experienced some of the 20 items. The percentages of patients experiencing each item and the mean levels of severity experienced are presented in Table D-1 in Additional file 4.

**Table 3:** Patient characteristics

Characteristic	Patients (*N* = 200)
Age (years), mean (SD)	42 (14)
Female	101 (51%)
Employed (full-time, part-time, or self-employed)	153 (77%)
Has had psoriasis for at least 1 year/5 years/10 years	148 (74%)/60 (30%)/29 (15%)
Has had psoriatic arthritis for at least 1 year/5 years/10 years	124 (62%)/34 (17%)/12 (6%)
More than five hand areas covered with psoriasis patches in the past week	88 (44%)
Mean (SD) rating for overall skin symptom rating in the past week (0, did not affect how I felt at all; 10 severely affected how I felt)	6.8 (2.5)
Mean (SD) rating for overall joint symptom rating in the past week (0, did not affect how I felt at all; 10 severely affected how I felt)	6.4 (2.6)
Percent ever using treatment for psoriasis or psoriatic arthritis (checked all that applied)	
Creams, lotions, ointments, foam	84
Oral prescription medicines	54
Light therapy	27
Injectable medicines	39
Infusions at the doctor’s office	17
Other	3
None of the above	1

Data presented as *n* (%) unless stated otherwise

*SD* standard deviation

Nielsen invited 10,791 individuals to be screened for eligibility for the physician study through email invitation. A total of 466 individuals accessed the survey screener, 396 (85% of those screened) were eligible, 394 (99%) consented to participate, and 300 (76%) completed the survey, comprising 150 rheumatologists and 150 dermatologists. Table D-2 in Additional file 4 presents characteristics of the 300 physicians who completed the survey. Physicians were also asked which of the 20 items their patients reported most and least often. The responses are reported separately for rheumatologists and dermatologists. Pearson’s chi-squared tests showed no difference between rheumatologists and dermatologists (at the 5% level) in their responses to most of the commonly reported skin and joint symptoms but a statistically significant difference for patient reporting of the impact of their symptoms on daily activities. For example, 59% of rheumatologists responded that the most commonly reported impact on daily activity was discomfort while doing everyday tasks compared with 33% for dermatologists. The percentages for the most and least reported items by patients to the rheumatologists and dermatologists are presented in Table D-3 in Additional file 4.

### Relative-bother weights

**Fig. 2:**
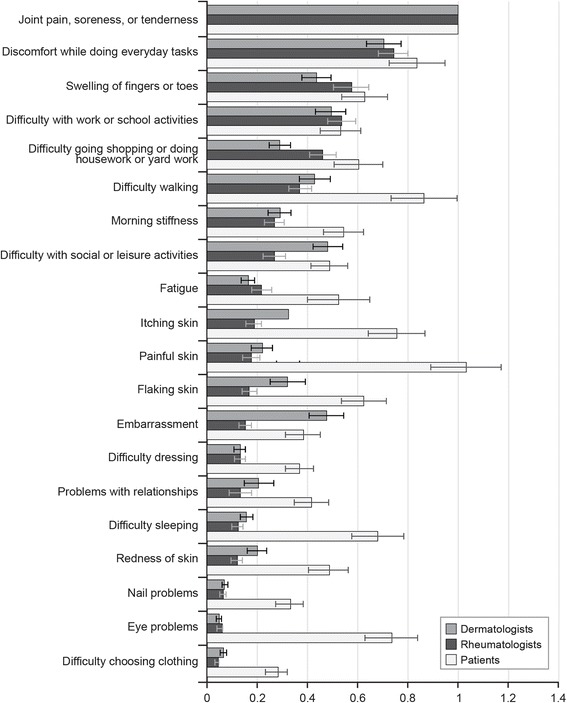
Best–worst scaling relative-bother estimates: dermatologists, rheumatologists, and patients (*N* = 500). Bars for each estimate indicate 95% confidence intervals

The bother of items was estimated relative to bother from joint pain, soreness, or tenderness, which was assigned a value of 1.00. Bother scores less than 1.00 indicated an item was less bothersome than joint pain, soreness, or tenderness; a score greater than 1 indicated that an item was more bothersome than joint pain, soreness, or tenderness. Relative-bother scores are presented in Fig. 2. Since the differences in responses for the three different patient types for both rheumatologists and dermatologists were mostly small and not statistically significant, Fig. 2 presents pooled data for the three patient types.

#### Physician specialty comparisons

Figure 2 allows comparison of bother estimates for physicians. Both types of physicians assessed joint pain, soreness, or tenderness as most bothersome and discomfort while doing everyday tasks as the next most bothersome. Furthermore, both assessed nail problems, eye problems, and difficulty choosing clothing as least bothersome. However, embarrassment, difficulty with social or leisure activities, and flaking skin and redness of skin were perceived relatively more bothersome compared with joint pain, soreness, or tenderness by dermatologists than by rheumatologists (*P* < 0.05). In contrast, rheumatologists perceived swelling of fingers and toes and difficulty shopping or doing housework or yard work as relatively more bothersome compared with joint pain, soreness, or tenderness than dermatologists (*P* < 0.05).

#### Patient and physician comparisons

Figure 2 also presents bother estimates for patients compared to physicians. For patients, the most bothersome item was painful, inflamed, or broken skin, followed closely by joint pain, soreness, or tenderness. The least bothersome item to patients was difficulty choosing clothing. Unlike physicians, patients were as bothered by painful, inflamed, or broken skin as they were by joint pain, soreness, or tenderness. Patients were also as bothered by difficulty walking as they were by discomfort while doing everyday tasks. Differences between patients and physicians suggest that while physicians may view a few key items as particularly bothersome and others as less so, patients view more of the items as almost equally bothersome.

There were also differences among patients, rheumatologists, and dermatologists in their assessment of the bother of items relative to joint pain, soreness, or tenderness when looking separately at skin symptoms, joint symptoms, and impact on daily activities. Tables E-1 and E-2 in Additional file 5 present the relative bother weights for patients and physicians, respectively, organized by item type. Painful, inflamed, or broken skin was the most bothersome of all the symptoms for patients. Among skin symptoms, dermatologists assessed embarrassment to be more bothersome relative to joint pain, soreness, or tenderness than did rheumatologists (*P* < 0.05); however, dermatologists’ assessment of embarrassment was similar to that of patients. Except for embarrassment, patients assessed all other skin symptoms as more bothersome relative to joint pain, soreness, or tenderness than did physicians (*P* < 0.05). Among joint symptoms, patients assessed difficulty walking, morning stiffness, fatigue, difficulty dressing, and eye problems to be more bothersome relative to joint pain, soreness, or tenderness than did either rheumatologists (*P* < 0.05) or dermatologists (*P* < 0.05). Swelling of fingers or toes was viewed as equally bothersome among patients and rheumatologists but less bothersome among dermatologists than among patients (*P* < 0.05) relative to joint pain, soreness, and tenderness. Finally, among impacts on daily activities, patients assessed difficulty sleeping and problems with relationships to be relatively more bothersome than did physicians (*P* < 0.05). Discomfort while doing everyday tasks and difficulty with work or school activities were viewed equally bothersome relative to joint pain, soreness, or tenderness across patients and physicians.

#### Patient subgroup comparisons

**Fig. 3:**
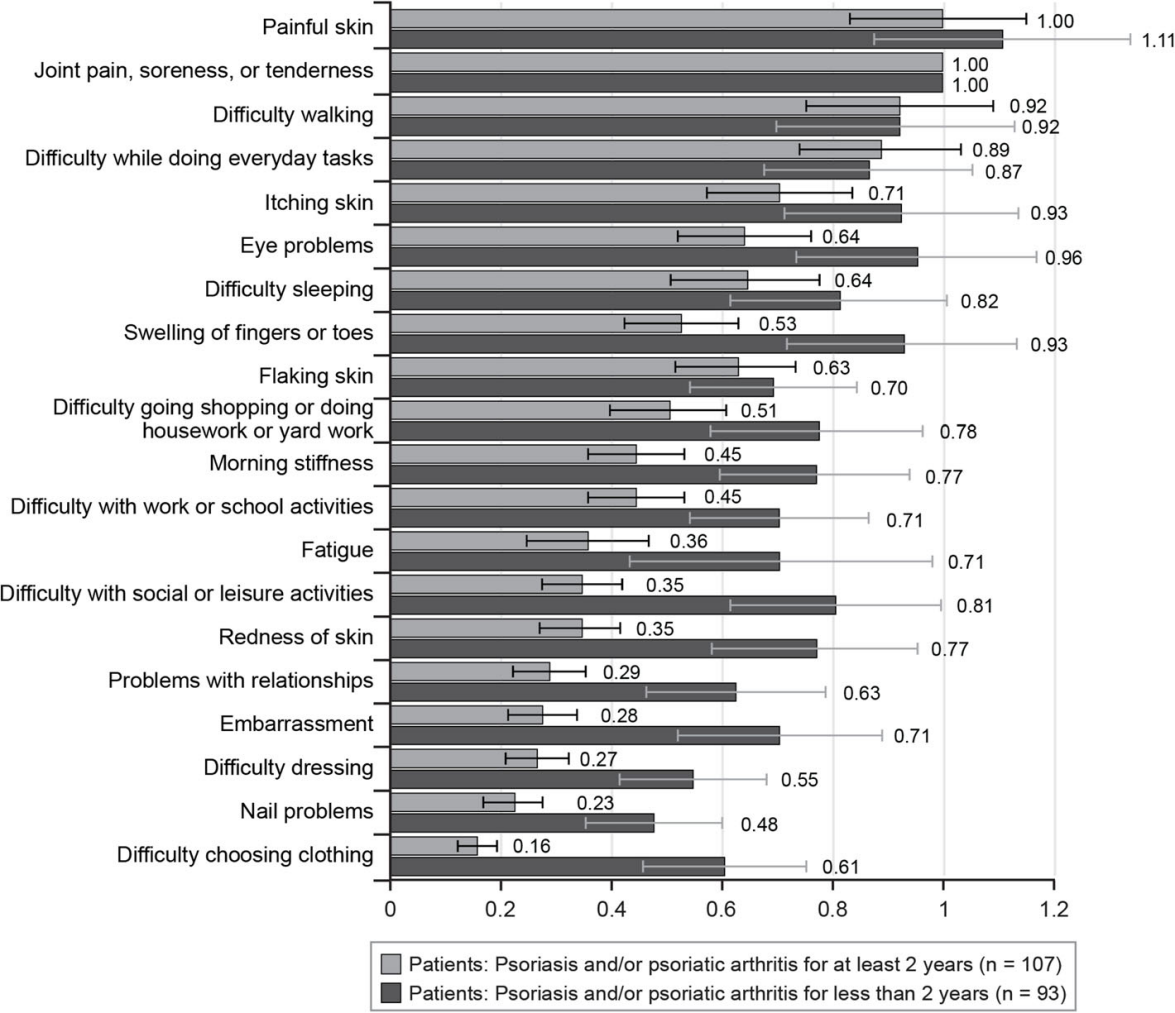
Best–worst scaling relative-bother estimates: time since diagnosis subgroups (*N* = 200). Bars for each estimate indicate 95% confidence intervals

On average, younger patients (< 38 years), those with more severe skin symptoms, and those recently diagnosed with psoriasis and/or psoriatic arthritis (< 2 years) had higher relative bother scores compared with joint pain, soreness, or tenderness for most items. Figures C-1–Figure C-11 in Additional file 3 present the results of all patient subgroup analyses. The magnitude of differences in relative bother scores between the patient subgroup pairs was greatest for time since diagnosis. The most bothersome items were the same for both subgroups. However, for patients who were diagnosed less than 2 years before the survey, many other items were significantly more bothersome relative to joint pain, soreness, or tenderness than for patients with a longer time since diagnosis (see Fig. 3).

## Discussion

Because patients with psoriatic disease commonly have both skin and joint symptoms, these symptoms ideally would be comanaged by both rheumatologists and dermatologists. Studies of chronic inflammatory diseases suggest that optimal treatment should rely on shared decision-making between patients and their physicians [27, 28]. Thus, it is important to assess the differing perceptions patients and physician specialists have about symptoms associated with various organs involved in a disease. We compared perceptions of relative bother of skin and joint symptoms and of activity impairments for patients, rheumatologists, and dermatologists in the US.

The most notable difference between the patients and physicians was that physicians assessed bother to patients highly for a few key items, while patients assessed more items as equally bothersome. Key items perceived as bothersome by the physicians were joint pain, soreness, or tenderness; discomfort while doing everyday tasks; and difficulty with work or school activities. These items were all rated > 0.50 relative to 1.00 for joint pain, soreness, or tenderness, while all other items in the survey received relative bother ratings of < 0.50. Patients rated these items highly as well, but also rated other items highly (> 0.50), including itching; flaking skin; painful, inflamed, or broken skin; difficulty walking; difficulty sleeping; eye problems; fatigue; difficulty going shopping or doing housework or yard work; and morning stiffness. Patients rated painful, inflamed, or broken skin as relatively more bothersome (1.03) than joint pain, soreness, or tenderness (1.0). This was in contrast to dermatologists and rheumatologists, who rated painful, inflamed, or broken skin at 0.22 and 0.17, respectively; much lower than their rating of joint pain, soreness of tenderness (1.0). Thus, these skin symptoms are equally as bothersome as the joint symptoms to patients but not to their physicians. In addition, there were differences between the physician specialties, with dermatologists being more aware of bother of flaking skin or red skin and associated impacts on embarrassment and social and leisure activities. While rheumatologists and dermatologists were equally aware of bother of joint symptoms and discomfort doing everyday tasks, rheumatologists were more aware of the bother of joint-related symptoms such as swelling of fingers and toes and difficulty shopping or doing housework or yard work.

These findings have important implications concerning the management and treatment of patients with psoriatic arthritis and psoriasis. First, both rheumatologists and dermatologists should query patients about the severity of their skin and joint symptoms and impact on their daily activities, rather than only on the symptoms related to their specialty. Our results suggest that treatment by both specialists should have as its goal improvement in all skin and joint symptoms for patients with psoriatic involvement of both organ systems, especially for patients for whom symptoms or impairment are most severe and/or most bothersome. Specifically, those more recently diagnosed with psoriatic arthritis rated most of the items in the survey as equally bothersome.

Results from our study are similar to results in two publications reporting results from a single study that used a different approach to assign patients’ relative importance weights for different symptoms of psoriatic arthritis [21, 22]. In that study, 12 patient research partners with psoriatic arthritis identified domains that had the greatest impact on their lives. A larger group of patients (*n* = 139) was then asked to assign 100 importance points to selected domains, and these distributions were used to develop relative rankings of the domains. The most important domains identified in that study (with at least 50% of patients considering it a priority) were pain in joints, spine, and skin; skin problems, including itching; fatigue, including being physically tired, mental fatigue, and lack of energy; and ability to work or perform other activities. Similar results were shown in our study for patients. However, corresponding values for physicians were different from those of patients in several cases in our study, including a lower rating of the relative bother of fatigue.

A study using a BWS approach, similar to the approach used in our study, for both physicians and patients with acute coronary syndromes [23] showed differences in relative importance of benefits and risks of antithrombotic therapy for cardiologists and patients. Our study and the study by Yuan et al. [23] indicate the need for physicians to take into account relative bother to patients of different disease manifestations as well as importance of benefits and risks of standard treatment regimens when prescribing treatment. This is necessary since patients’ perceptions may differ from physicians’ perceptions.

This study has several limitations. One inherent limitation is that the respondents evaluated hypothetical combinations of psoriatic arthritis and psoriasis symptoms and functional limitations, and their choices do not have the same significance as evaluations of actual symptoms.

As in any survey research study, sample representativeness may be a potential study limitation. Patients self-reported their physician diagnosis of psoriasis and psoriatic arthritis. It is possible that some patients may not have received this diagnosis. Another limitation was that the sample was small relative to the population invited to participate. It is, therefore, difficult to determine how representative our sample of patients was or whether our results are generalizable to all patients with psoriasis and psoriatic arthritis in the US. The participants’ self-reported previous use of injectable or infused drugs was higher than that of patients with psoriasis (~ 15%) from a US database study [29], which would be expected for those with psoriatic arthritis.

Finally, one must use caution in the interpretation of the study results. They should not be used to indicate the level of importance of individual symptoms, but rather the relative importance of each symptom compared to the other symptoms included in the study. Nevertheless, the survey items were chosen to reflect symptoms and limitations that patients have reported as important to them.

In this study, we have demonstrated that rheumatologists and dermatologists may differ in their assessment of bother of skin symptoms. There are multiple potential reasons for the differences between specialists, including physicians’ need to focus on a subset of psoriatic manifestations given time limitations of single appointments or differences in symptoms mentioned by patients to each type of physician involved in their psoriatic disease care. Nevertheless, the greater perception of the bother of skin symptoms by dermatologists and their lesser perception of the bother of some of the joint-related symptoms might partially explain the observed delay in referral of their patients for assessment by a rheumatologist [6, 7].

## Conclusions

Our results also showed that both specialists may differ from patients in their assessment of bother of the manifestations of psoriatic arthritis. This suggests the need for both rheumatologists and dermatologists to ask their patients about the bother of all manifestations of psoriatic disease to ensure optimal drug treatment along with other patient management options. Drug treatments available for both psoriatic arthritis and psoriasis have shown efficacy in alleviating both skin and joint symptoms. Since both types of symptoms are considered equally bothersome by patients with psoriatic arthritis and psoriasis, treatment regimens should be selected that can alleviate both types of symptoms, whichever type of specialist is treating the patient. In addition, efficacy of the treatment regimen for relief of both skin and joint symptoms and functional limitations should be carefully monitored, keeping in mind that although persistent skin inflammation can be reversed, joint damage cannot.

## Additional files

Additional file 1: Survey instrument administered to patients (PDF 298 kb)

Additional file 2: Survey instrument administered to rheumatologists and dermatologists (PDF 189 kb)

Additional file 3: Results of patient subgroup analyses (DOCX 1800 kb)

Additional file 4: Patients’ experience with survey items and physicians’ medical experience and experience with survey items. (DOCX 45 kb)

Additional file 5: Relative-bother estimates for patients and physicians, grouped by survey item type. (DOCX 31 kb)

## Abbreviations

BWS: Best–worst scaling
RBW: Relative-bother weight
RPL: Random-parameters logit
SD: Standard deviation
US: United States
