# Supplementary material for: Comparison of US patient, rheumatologist, and dermatologist perceptions of psoriatic disease symptoms: results from the DISCONNECT study
Source: Arthritis Res Ther. 2018 May 31;20:102. doi: 10.1186/s13075-018-1601-4 (PMC5977464; doi:10.1186/s13075-018-1601-4)
Supplement: Supplementary file 1 — Survey instrument administered to patients (PDF 298 kb) [file 13075_2018_1601_MOESM1_ESM.pdf]

Psoriasis and Psoriatic Arthritis Survey

Thank you for agreeing to take this survey about psoriasis and psoriatic arthritis. First, we would like to ask you a few questions about your experience with psoriasis and psoriatic arthritis.

1. How long ago did a doctor tell you that you have psoriasis?

- ☐ Less than 6 months
- ☐ 6 months to less than 1 year
- ☐ 1 year to less than 2 years
- ☐ 2 years to less than 5 years
- ☐ 5 years to less than 10 years
- ☐ 10 years or more

2. How long ago did a doctor tell you that you have psoriatic arthritis?

- ☐ Less than 6 months
- ☐ 6 months to less than 1 year
- ☐ 1 year to less than 2 years
- ☐ 2 years to less than 5 years
- ☐ 5 years to less than 10 years
- ☐ 10 years or more

3. Where on your body have you had psoriasis patches in the past week? *(Check all that apply.)*

- ☐ Face
- ☐ Neck
- ☐ Scalp
- ☐ Genital area
- ☐ Hands
- ☐ Feet
- ☐ Fingernails
- ☐ Toenails
- ☐ Arms (includes elbows)
- ☐ Legs (includes hips and knees)
- ☐ Chest or back (includes stomach)
- ☐ Buttocks
- ☐ Other
- ☐ I have not had any psoriasis patches in the past week

The amount of body area affected by psoriasis varies from person to person. The area of your hand, both palm and fingers, is about 1% of your total body area.

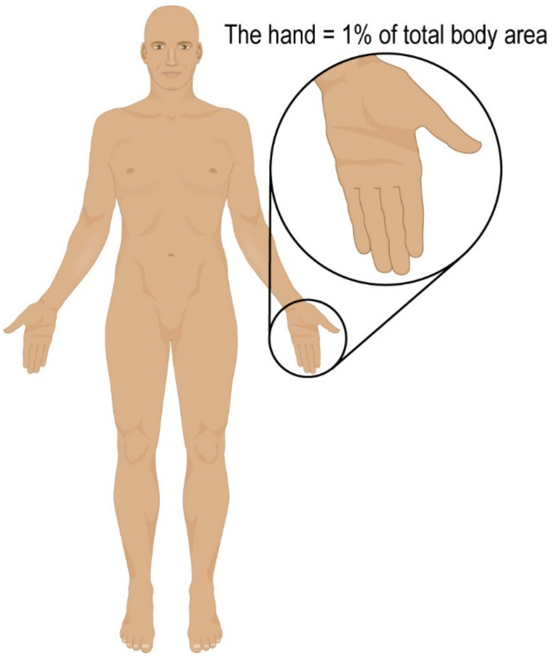

4. About how much of your body area has been covered with psoriasis patches <u>in the past week</u>?

- ☐ 2 hand areas or less
- ☐ 3 to 5 hand areas
- ☐ 6 to 9 hand areas
- ☐ 10 to 14 hand areas
- ☐ 15 to 19 hand areas
- ☐ More than 20 hand areas
- ☐ I have not had any psoriasis patches in the past week

5. In which of the following places on your body have you had pain, swelling, or stiffness <u>in the past week</u> because of your psoriatic arthritis?

(Check all that apply.)

- ☐ In your fingers
- ☐ In your toes
- ☐ In your feet
- ☐ In your ankles
- ☐ In your knees
- ☐ In your hips
- ☐ In your elbows
- ☐ In your neck
- ☐ In your upper back
- ☐ In your lower back
- ☐ In your shoulders
- ☐ In your buttocks
- ☐ None of the above

6. What treatments have you ever used to treat your psoriasis and psoriatic arthritis? *(Check all that apply.)*

- ☐ Creams, lotions, ointments, or foams (includes prescription and over-the-counter treatments)
- ☐ Shampoos
- ☐ Oral prescription medicines
- ☐ Light therapy
- ☐ Injectable medicines
- ☐ Intravenous infusions at a doctor's office, infusion center, or hospital
- ☐ Other
- ☐ None of the above

7. [If at least one treatment (other than "Other" or "None of the above") was selected in Q6] What treatments are you currently using to treat your psoriasis and psoriatic arthritis? *(Check all that apply.)*

- ☐ Creams, lotions, ointments, or foams (includes prescription and over-the-counter treatments) [include only if selected in Q6]
- ☐ Shampoos [include only if selected in Q6]
- ☐ Oral prescription medicines [include only if selected in Q6]
- ☐ Light therapy [include only if selected in Q6]
- ☐ Injectable medicines [include only if selected in Q6]
- ☐ Intravenous infusions at a doctor's office, infusion center, or hospital [include only if selected in Q6]
- ☐ Other
- ☐ None of the above

8. [If creams, lotions, ointments, or foams selected in Q7] How long have you been using the creams, lotions, ointments, or foams that you are currently using?

- ☐ Less than 6 months
- ☐ At least 6 months, but less than 1 year
- ☐ At least 1 year, but less than 2 years
- ☐ At least 2 years, but less than 5 years
- ☐ 5 years or more

9. [If shampoos selected in Q7] How long have you been using the shampoos that you are currently using?

- ☐ Less than 6 months
- ☐ At least 6 months, but less than 1 year
- ☐ At least 1 year, but less than 2 years
- ☐ At least 2 years, but less than 5 years
- ☐ 5 years or more

10. [If oral prescription medicines selected in Q7] How long have you been using the oral prescription medicines that you are currently using?

- ☐ Less than 6 months
- ☐ At least 6 months, but less than 1 year
- ☐ At least 1 year, but less than 2 years
- ☐ At least 2 years, but less than 5 years
- ☐ 5 years or more

11. [If light therapy selected in Q7] How long have you been using light therapy?

- ☐ Less than 6 months
- ☐ At least 6 months, but less than 1 year
- ☐ At least 1 year, but less than 2 years
- ☐ At least 2 years, but less than 5 years
- ☐ 5 years or more

12. [If injectable medicines selected in Q7] How long have you been taking the injectable medicines that you are currently taking?

- ☐ Less than 6 months
- ☐ At least 6 months, but less than 1 year
- ☐ At least 1 year, but less than 2 years
- ☐ At least 2 years, but less than 5 years
- ☐ 5 years or more

13. [If intravenous infusions selected in Q7] How long have you been receiving the intravenous infusions that you are currently receiving?

- ☐ Less than 6 months
- ☐ At least 6 months, but less than 1 year
- ☐ At least 1 year, but less than 2 years
- ☐ At least 2 years, but less than 5 years
- ☐ 5 years or more

Psoriasis and Psoriatic Arthritis

Psoriasis causes patches of scaly, red, or white skin called *plaques*. Psoriatic arthritis causes joint swelling and pain that can lead to permanent damage. Inflammation happens with both psoriasis and psoriatic arthritis because your immune system attacks your body instead of something outside of it. These two conditions are often related, but you may have skin problems and joint problems in different parts of your body.

Please go to the next page.

Symptoms of Psoriasis and Psoriatic Arthritis

The next few pages of this survey will describe different symptoms of psoriasis and psoriatic arthritis, including potential impacts on your daily activities. These symptoms are presented in three groups: skin symptoms, joint symptoms, and the impact of these conditions on your daily activities. This information will help you answer questions later in the survey. You can refer back to this information as you take the survey.

Please go to the next page.

Skin Symptoms

Symptom	Description
Itching skin	Physically irritated skin resulting in the urge to scratch
Redness of skin	Red or salmon-pink color of psoriasis-affected skin
Flaking skin	Skin shedding
Painful skin	Painful inflamed or broken skin
Nail problems	Discoloration or pitting of the fingernails or toenails or separation of the nail from the nail bed
Difficulty choosing clothing	Skin problems influencing the clothing you wear
Embarrassment	Being embarrassed or self-conscious because of your skin

14. Please indicate which of the following skin symptoms you have <u>ever</u> experienced. *(Check all that apply.)*

- ☐ Itching skin
- ☐ Redness of skin
- ☐ Flaking skin
- ☐ Painful skin
- ☐ Nail problems
- ☐ Difficulty choosing clothing
- ☐ Embarrassment

15. [If at least one symptom was selected in Q14] Please indicate which of the following skin symptoms you have experienced <u>in the past week</u>.
(Check all that apply.)

- ☐ Itching skin [If selected in Q14]
- ☐ Redness of skin [If selected in Q14]
- ☐ Flaking skin [If selected in Q14]
- ☐ Painful skin [If selected in Q14]
- ☐ Nail problems [If selected in Q14]
- ☐ Difficulty choosing clothing [If selected in Q14]
- ☐ Embarrassment [If selected in Q14]
- ☐ I have not had any of these skin symptoms in the past week [Can only select this response if no other response to the question; if selected, skip to next section “Joint symptoms”]

16. [If at least one symptom was selected in Q15] Select the number that best describes <u>how severe</u> each of your skin symptoms has been <u>in the past week</u>:

Itching skin [If selected in Q15]

None	0	1	2	3	4	5	6	7	8	9	10	Extreme

Redness of skin [If selected in Q15]

None	0	1	2	3	4	5	6	7	8	9	10	Extreme

Flaking skin [If selected in Q15]

None	0	1	2	3	4	5	6	7	8	9	10	Extreme

Painful skin [If selected in Q15]

None	0	1	2	3	4	5	6	7	8	9	10	Extreme

Nail problems [If selected in Q15]

None	0	1	2	3	4	5	6	7	8	9	10	Extreme

Difficulty choosing clothing [If selected in Q15]

No difficulty	0	1	2	3	4	5	6	7	8	9	10	Extreme difficulty

Embarrassment [If selected in Q15]

No difficulty	0	1	2	3	4	5	6	7	8	9	10	Extreme difficulty

17. Select the number that best describes how much your skin symptoms have affected you overall <u>in the past week</u>.

Did not affect how I felt at all	0	1	2	3	4	5	6	7	8	9	10	Severely affected how I felt

Joint Symptoms

Symptom	Description
Joint pain, soreness, or tenderness	Stiffness, pain, throbbing, swelling, and tenderness in one or more joints
Swelling of fingers or toes	Sausage-like swelling of one or more fingers or toes
Fatigue	Tiredness and lack of energy that doesn’t go away with sleep
Morning stiffness	Stiffness after resting that makes it difficult to move your joints
Eye problems	Eye swelling, redness in or around your eyes, eye pain, and/or blurry vision
Difficulty dressing	Difficulty tying shoelaces and buttoning your clothes
Difficulty walking	Difficulty walking at a normal speed

18. Please indicate which of the following joint symptoms you have ever experienced. (*Check all that apply.*)

- ☐ Joint pain, soreness, or tenderness
- ☐ Swelling of fingers or toes
- ☐ Fatigue
- ☐ Morning stiffness
- ☐ Eye problems
- ☐ Difficulty dressing
- ☐ Difficulty walking

19. [If at least one symptom was selected in Q18] Please indicate which of the following joint symptoms you have experienced <u>in the past week</u>. (*Check all that apply.*)

- ☐ Joint pain, soreness, or tenderness [If selected in Q18]
- ☐ Swelling of fingers or toes [If selected in Q18]
- ☐ Fatigue [If selected in Q18]
- ☐ Morning stiffness [If selected in Q18]
- ☐ Eye problems [If selected in Q18]
- ☐ Difficulty dressing [If selected in Q18]
- ☐ Difficulty walking [If selected in Q18]
- ☐ I have not had any of these joint symptoms in the past week [Can only select this response if no other response to the question; if selected, skip to next section “Impact of Psoriasis and Psoriatic Arthritis on Daily Activities”]

20. [If at least one symptom was selected in Q19] Select the number that best describes how severe each of your joint symptoms has been in the past week:

Joint pain, soreness, or tenderness [If selected in Q19]

None	0	1	2	3	4	5	6	7	8	9	10	Extreme

Swelling of fingers or toes [If selected in Q19]

None	0	1	2	3	4	5	6	7	8	9	10	Extreme

Fatigue [If selected in Q19]

None	0	1	2	3	4	5	6	7	8	9	10	Extreme

Morning stiffness [If selected in Q19]

None	0	1	2	3	4	5	6	7	8	9	10	Extreme

Eye problems [If selected in Q19]

None	0	1	2	3	4	5	6	7	8	9	10	Extreme

Difficulty dressing [If selected in Q19]

No difficulty	0	1	2	3	4	5	6	7	8	9	10	Extreme difficulty

Difficulty walking [If selected in Q19]

No difficulty	0	1	2	3	4	5	6	7	8	9	10	Extreme difficulty

21. Select the number that best describes how much your joint symptoms have affected you overall in the past week.

Did not affect how I felt at all	0	1	2	3	4	5	6	7	8	9	10	Severely affected how I felt

Impact of Psoriasis and Psoriatic Arthritis on Daily Activities

Impact on Daily Activities	Description
Difficulty with work or school activities	Difficulty doing your normal work or schoolwork because of your psoriasis or psoriatic arthritis
Difficulty with social or leisure activities	Difficulty doing your normal social or leisure activities because of your psoriasis or psoriatic arthritis
Difficulty going shopping or doing housework or yard work	Difficulty going shopping or looking after your home or yard because of your psoriasis or psoriatic arthritis
Difficulty sleeping	Having poor sleep quality or sleep interruptions because of your psoriasis or psoriatic arthritis
Discomfort while doing everyday tasks	Discomfort doing everyday tasks, such as eating, bathing, or going to the bathroom, because of your psoriasis or psoriatic arthritis
Problems with relationships	Problems with partner, close friends, or family because of your psoriasis or psoriatic arthritis

22. Please indicate which of the following impacts on daily activities that you have ever experienced. *(Check all that apply.)*

- ☐ Difficulty with work or school activities
- ☐ Difficulty with social or leisure activities
- ☐ Difficulty going shopping or doing housework or yard work
- ☐ Difficulty sleeping
- ☐ Discomfort while doing everyday tasks
- ☐ Problems with relationships
- ☐ I have never experienced any of these impacts on my daily activities [Can only select this response if no other response to the question; if selected, skip to next section “Burden of Symptoms of Psoriasis and Psoriatic Arthritis”]

23. [If at least one impact was selected in Q22] Please indicate which of the following impacts on daily activities you have experienced <u>in the past week</u>.
(Check all that apply.)

- ☐ Difficulty with work or school activities [If selected in Q22]
- ☐ Difficulty with social or leisure activities [If selected in Q22]
- ☐ Difficulty going shopping or doing housework or yard work [If selected in Q22]
- ☐ Difficulty sleeping [If selected in Q22]
- ☐ Discomfort while doing everyday tasks [If selected in Q22]
- ☐ Problems with relationships [If selected in Q22]
- ☐ I have not had difficulty with any daily activities in the past week [Can only select this response if no other response to the question; if selected, skip to next section “Burden of Symptoms of Psoriasis and Psoriatic Arthritis”]

24. [If at least one impact was selected in Q23] Select the number that best describes how severe each of the impacts on daily living has been in the past week:

Difficulty with work or school activities [If selected in Q23]

No difficulty	0	1	2	3	4	5	6	7	8	9	10	Extreme difficulty

Difficulty with social or leisure activities [If selected in Q23]

No difficulty	0	1	2	3	4	5	6	7	8	9	10	Extreme difficulty

Difficulty going shopping or doing housework or yard work [If selected in Q23]

No difficulty	0	1	2	3	4	5	6	7	8	9	10	Extreme difficulty

Difficulty sleeping [If selected in Q23]

No difficulty	0	1	2	3	4	5	6	7	8	9	10	Extreme difficulty

Discomfort while doing everyday tasks [If selected in Q23]

No discomfort	0	1	2	3	4	5	6	7	8	9	10	Extreme discomfort

Problems with relationships [If selected in Q23]

No problems	0	1	2	3	4	5	6	7	8	9	10	Extreme problems

Burden of Symptoms of Psoriasis and Psoriatic Arthritis

We are interested in knowing how bothered you would be by each of the symptoms described earlier in this survey.

Even if you are not currently experiencing these symptoms or have never experienced these symptoms in the past, we would like to know how bothered you would be if you experienced these symptoms.

The table below shows 5 possible symptoms. Please tell us which one would bother you the most. In other words, which one would you want to avoid the most?

BWS Question 1

I would be bothered by this the MOST (Check only ONE)	Symptom
☐	Problems with relationships
☐	Joint pain, soreness, or tenderness
☐	Difficulty with social or leisure activities
☐	Redness of skin
☐	Difficulty sleeping

25. In the previous question, you told us that [insert symptom chosen in previous question] was the most bothersome symptom.

That means that you think that [insert symptom chosen in previous question] is more bothersome than ______, ______, ______, and ______ [insert 4 symptoms not chosen in previous question].

Is that correct?

- ☐ Yes
- ☐ No

[If yes, go to next page.]

[If no, show previous BWS question with the following heading: "Please indicate which of these 5 symptoms would bother you the most by checking the box to the left of the symptom."]

[Do not allow respondents to select the feature checked in the previous question.]

Now, please tell us which of these same 5 symptoms bothers you the least. In other words, which one would you choose if you had to experience one of these symptoms?

Symptom	I would be bothered by this the LEAST (Check only ONE)
Problems with relationships	☐
Joint pain, soreness, or tenderness	☐
Difficulty with social or leisure activities	☐
Redness of skin	☐
Difficulty sleeping	☐

26. In the previous question, you told us that [insert symptom chosen in previous question] was the least bothersome symptom.

That means that you think that [insert symptom chosen in previous question] is less bothersome than ______, ______, ______, and ____ [insert 4 symptoms not chosen in previous question].

Is that correct?

- ☐ Yes
- ☐ No

[If yes, go to next page.]

[If no, show previous BWS question with the following heading: "Please indicate which of these 5 symptoms would bother you the least by checking the box to the right of the symptom."]

For each of the next 15 questions, we will show you a set of 5 symptoms.

For each set of symptoms, please select the symptom that would bother you the <u>most</u> by checking the box to the <u>left</u> of the symptom.

Then, please select the symptom that would bother you the <u>least</u> by checking the box to the <u>right</u> of that symptom.

Please choose only 1 symptom as the one that would bother you the most and 1 symptom that would bother you the least in each question.

BWS Question 2

Please indicate which one of the 5 symptoms listed below would bother you the most and which one would bother you the least if you experienced these symptoms.

I would be bothered by this the MOST (Check only ONE)	Symptom	I would be bothered by this the LEAST (Check only ONE)
☐	Redness of skin	☐
☐	Swelling of fingers or toes	☐
☐	Itching skin	☐
☐	Painful skin	☐
☐	Difficulty going shopping or doing housework or yard work	☐

BWS Question 3

Please indicate which one of the 5 symptoms listed below would bother you the most and which one would bother you the least if you experienced these symptoms.

I would be bothered by this the MOST (Check only ONE)	Symptom	I would be bothered by this the LEAST (Check only ONE)
☐	Joint pain, soreness, or tenderness	☐
☐	Morning stiffness	☐
☐	Difficulty with work or school activities	☐
☐	Flaking skin	☐
☐	Discomfort while doing everyday tasks	☐

BWS Question 4

Please indicate which one of the 5 symptoms listed below would bother you the most and which one would bother you the least if you experienced these symptoms.

I would be bothered by this the MOST (Check only ONE)	Symptom	I would be bothered by this the LEAST (Check only ONE)
☐	Discomfort while doing everyday tasks	☐
☐	Difficulty going shopping or doing housework or yard work	☐
☐	Difficulty dressing	☐
☐	Difficulty choosing clothing	☐
☐	Difficulty walking	☐

BWS Question 5

Please indicate which one of the 5 symptoms listed below would bother you the most and which one would bother you the least if you experienced these symptoms.

I would be bothered by this the MOST (Check only ONE)	Symptom	I would be bothered by this the LEAST (Check only ONE)
☐	Flaking skin	☐
☐	Itching skin	☐
☐	Morning stiffness	☐
☐	Embarrassment	☐
☐	Nail problems	☐

BWS Question 6

Please indicate which one of the 5 symptoms listed below would bother you the most and which one would bother you the least if you experienced these symptoms.

I would be bothered by this the MOST (Check only ONE)	Symptom	I would be bothered by this the LEAST (Check only ONE)
☐	Difficulty choosing clothing	☐
☐	Nail problems	☐
☐	Problems with relationships	☐
☐	Eye problems	☐
☐	Painful skin	☐

BWS Question 7

Please indicate which one of the 5 symptoms listed below would bother you the most and which one would bother you the least if you experienced these symptoms.

I would be bothered by this the MOST (Check only ONE)	Symptom	I would be bothered by this the LEAST (Check only ONE)
☐	Difficulty dressing	☐
☐	Difficulty with social or leisure activities	☐
☐	Difficulty walking	☐
☐	Fatigue	☐
☐	Embarrassment	☐

BWS Question 8

Please indicate which one of the 5 symptoms listed below would bother you the most and which one would bother you the least if you experienced these symptoms.

I would be bothered by this the MOST (Check only ONE)	Symptom	I would be bothered by this the LEAST (Check only ONE)
☐	Difficulty sleeping	☐
☐	Fatigue	☐
☐	Swelling of fingers or toes	☐
☐	Difficulty with work or school activities	☐
☐	Eye problems	☐

BWS Question 9

Please indicate which one of the 5 symptoms listed below would bother you the most and which one would bother you the least if you experienced these symptoms.

I would be bothered by this the MOST (Check only ONE)	Symptom	I would be bothered by this the LEAST (Check only ONE)
☐	Painful skin	☐
☐	Difficulty dressing	☐
☐	Flaking skin	☐
☐	Joint pain, soreness, or tenderness	☐
☐	Fatigue	☐

BWS Question 10

Please indicate which one of the 5 symptoms listed below would bother you the most and which one would bother you the least if you experienced these symptoms.

I would be bothered by this the MOST (Check only ONE)	Symptom	I would be bothered by this the LEAST (Check only ONE)
☐	Eye problems	☐
☐	Flaking skin	☐
☐	Difficulty walking	☐
☐	Problems with relationships	☐
☐	Itching skin	☐

BWS Question 11

Please indicate which one of the 5 symptoms listed below would bother you the most and which one would bother you the least if you experienced these symptoms.

I would be bothered by this the MOST (Check only ONE)	Symptom	I would be bothered by this the LEAST (Check only ONE)
☐	Difficulty going shopping or doing housework or yard work	☐
☐	Difficulty with work or school activities	☐
☐	Painful skin	☐
☐	Difficulty with social or leisure activities	☐
☐	Nail problems	☐

BWS Question 12

Please indicate which one of the 5 symptoms listed below would bother you the most and which one would bother you the least if you experienced these symptoms.

I would be bothered by this the MOST (Check only ONE)	Symptom	I would be bothered by this the LEAST (Check only ONE)
☐	Swelling of fingers or toes	☐
☐	Discomfort while doing everyday tasks	☐
☐	Embarrassment	☐
☐	Problems with relationships	☐
☐	Joint pain, soreness, or tenderness	☐

BWS Question 13

Please indicate which one of the 5 symptoms listed below would bother you the most and which one would bother you the least if you experienced these symptoms.

I would be bothered by this the MOST (Check only ONE)	Symptom	I would be bothered by this the LEAST (Check only ONE)
☐	Morning stiffness	☐
☐	Redness of skin	☐
☐	Eye problems	☐
☐	Swelling of fingers or toes	☐
☐	Difficulty with social or leisure activities	☐

BWS Question 14

Please indicate which one of the 5 symptoms listed below would bother you the most and which one would bother you the least if you experienced these symptoms.

I would be bothered by this the MOST (Check only ONE)	Symptom	I would be bothered by this the LEAST (Check only ONE)
☐	Difficulty walking	☐
☐	Embarrassment	☐
☐	Difficulty choosing clothing	☐
☐	Redness of skin	☐
☐	Difficulty with work or school activities	☐

BWS Question 15

Please indicate which one of the 5 symptoms listed below would bother you the most and which one would bother you the least if you experienced these symptoms.

I would be bothered by this the MOST (Check only ONE)	Symptom	I would be bothered by this the LEAST (Check only ONE)
☐	Fatigue	☐
☐	Difficulty choosing clothing	☐
☐	Difficulty going shopping or doing housework or yard work	☐
☐	Morning stiffness	☐
☐	Difficulty sleeping	☐

BWS Question 16

Please indicate which one of the 5 symptoms listed below would bother you the most and which one would bother you the least if you experienced these symptoms.

I would be bothered by this the MOST (Check only ONE)	Symptom	I would be bothered by this the LEAST (Check only ONE)
☐	Nail problems	☐
☐	Difficulty sleeping	☐
☐	Discomfort while doing everyday tasks	☐
☐	Difficulty dressing	☐
☐	Itching skin	☐

Other Questions About You

27. In what year were you born? ___________

28. What is your gender?

- ☐ Female
- ☐ Male
- ☐ Prefer not to answer

29. How would you describe your race or ethnicity? (*Check all that apply.*)

- ☐ White or Caucasian
- ☐ Black or African American
- ☐ Asian
- ☐ Hispanic or Latino
- ☐ Native Hawaiian or other Pacific Islander
- ☐ American Indian or Alaska Native
- ☐ Other
- ☐ Prefer not to answer

30. What is your marital status?

- ☐ Single/never married
- ☐ Married/living as married/civil partnership
- ☐ Divorced or separated
- ☐ Widowed/surviving partner
- ☐ Other
- ☐ Prefer not to answer

31. What is the highest level of education you have completed?

- ☐ Less than high school
- ☐ Some high school
- ☐ High school or equivalent (e.g., GED)
- ☐ Some college but no degree
- ☐ Technical school
- ☐ Associate's degree (2-year college degree)
- ☐ 4-year college degree (e.g., BA, BS)
- ☐ Some graduate school but no degree
- ☐ Graduate or professional degree (e.g., MBA, MS, MD, PhD)

32. Which of the following best describes your employment status?

- ☐ Employed full time
- ☐ Employed part time
- ☐ Self-employed
- ☐ Homemaker
- ☐ Student
- ☐ Retired
- ☐ Disabled/unable to work
- ☐ On medical leave of absence from work
- ☐ Unemployed but looking for work
- ☐ Unemployed and not looking for work

33. What type of health insurance do you have? (*Check all that apply.*)

- ☐ I do not have health insurance
- ☐ Private insurance that I pay for myself
- ☐ Private insurance that my or my spouse’s employer pays all or part of
- ☐ Medicaid
- ☐ Medicare
- ☐ Veterans Health insurance
- ☐ Other
- ☐ Don’t know/not sure

34. What was your total household income before tax and other deductions in 2015?

- ☐ Less than $20,000
- ☐ $20,000 to $29,999
- ☐ $30,000 to $39,999
- ☐ $40,000 to $49,999
- ☐ $50,000 to $59,999
- ☐ $60,000 to $69,999
- ☐ $70,000 to $79,999
- ☐ $80,000 to $89,999
- ☐ $90,000 to $99,999
- ☐ $100,000 to $149,999
- ☐ $150,000 to $199,999
- ☐ $200,000 or more
- ☐ Don’t know/not sure
- ☐ Prefer not to answer
