# Supplementary material for: Comparison of US patient, rheumatologist, and dermatologist perceptions of psoriatic disease symptoms: results from the DISCONNECT study
Source: Arthritis Res Ther. 2018 May 31;20:102. doi: 10.1186/s13075-018-1601-4 (PMC5977464; doi:10.1186/s13075-018-1601-4)
Supplement: Supplementary file 2 — Survey instrument administered to rheumatologists and dermatologists (PDF 189 kb) [file 13075_2018_1601_MOESM2_ESM.pdf]

Psoriasis and Psoriatic Arthritis Survey

Thank you for agreeing to take this survey about psoriasis and psoriatic arthritis. In this survey, we will ask for your professional opinion regarding symptoms and outcomes of plaque psoriasis and psoriatic arthritis.

1. How many years have you been in practice since completing your medical training?

- ☐ Less than 1 year
- ☐ 1-3 years
- ☐ 4-6 years
- ☐ 7-9 years
- ☐ 10-15 years
- ☐ 16-20 years
- ☐ 21-25 years
- ☐ More than 25 years

2. Which of the following describes your practice? (*Check all that apply.*)

- ☐ Office-based private practice
- ☐ Hospital-based private practice
- ☐ Academic hospital-based practice
- ☐ Other

4. On average, how many patients with plaque psoriasis do you treat each month?

- ☐ 5 patients or fewer
- ☐ 6-10 patients
- ☐ 11-20 patients

- ☐ 21-30 patients
- ☐ 31-40 patients
- ☐ 41-50 patients
- ☐ More than 50 patients

5. On average, how many patients with psoriatic arthritis do you treat each month?

- ☐ 5 patients or fewer
- ☐ 6-10 patients
- ☐ 11-20 patients
- ☐ 21-30 patients
- ☐ 31-40 patients
- ☐ 41-50 patients
- ☐ More than 50 patients

6. For what percentage of your patients do you prescribe biologic agents to treat psoriasis or psoriatic arthritis?

- ☐ I do not prescribe biologic agents
- ☐ More than 0%, but less than 25%
- ☐ 26%-50%
- ☐ 51%-75%
- ☐ 76%-100%

[if Q6 ≠ “I do not prescribe biologic agents”]

7. How long have you been prescribing biologic agents to treat patients with psoriasis or psoriatic arthritis?

- ☐ Less than 1 year
- ☐ 1-2 years
- ☐ 3-5 years
- ☐ More than 5 years

Symptoms of Psoriasis and Psoriatic Arthritis

The next few pages of this survey will describe different symptoms of psoriasis and psoriatic arthritis, including the potential impact of psoriasis and psoriatic arthritis on patients' daily activities. These symptoms are presented in three groups: symptoms of psoriasis, symptoms of psoriatic arthritis, and the impact of psoriasis and psoriatic arthritis on daily activities.

Symptoms of Psoriasis

Symptom	Description
Itching skin	Physically irritated skin resulting in the urge to scratch
Redness of skin	Red or salmon-pink color of psoriasis-affected skin
Flaking skin	Skin shedding
Painful skin	Painful inflamed or broken skin
Nail problems	Discoloration or pitting of the fingernails or toenails or separation of the nail from the nail bed
Difficulty choosing clothing	Skin problems influencing the clothing a patient wears
Embarrassment	Patient being embarrassed or self-conscious because of skin

8. Please indicate which of the following symptoms of psoriasis your patients with psoriasis or psoriatic arthritis report most often. *(Please check only one.)*

- ☐ Itching skin
- ☐ Redness of skin
- ☐ Flaking skin
- ☐ Painful skin
- ☐ Nail problems
- ☐ Difficulty choosing clothing
- ☐ Embarrassment

9. Please indicate which of the following symptoms of psoriasis your patients with psoriasis or psoriatic arthritis report least often. *(Please check only one.)*

- ☐ Itching skin [If not selected in Q7]
- ☐ Redness of skin [If not selected in Q7]
- ☐ Flaking skin [If not selected in Q7]
- ☐ Painful skin [If not selected in Q7]
- ☐ Nail problems [If not selected in Q7]
- ☐ Difficulty choosing clothing [If not selected in Q7]
- ☐ Embarrassment [If not selected in Q7]

Symptoms of Psoriatic Arthritis

Symptom	Description
Joint pain, soreness, or tenderness	Stiffness, pain, throbbing, swelling, or tenderness in one or more joints
Swelling of fingers or toes	Sausage-like swelling in one or more fingers or toes
Fatigue	Tiredness and lack of energy that doesn’t go away with sleep
Morning stiffness	Stiffness after resting that makes it difficult to move joints
Eye problems	Eye swelling, redness in or around eyes, eye pain, and/or blurry vision
Difficulty dressing	Difficulty tying shoelaces and buttoning clothes
Difficulty walking	Difficulty walking at a normal speed

10. Please indicate which of the following symptoms of psoriatic arthritis your patients with psoriasis or psoriatic arthritis report <u>most often</u>. (*Please check only one.*)

☐ Joint pain, soreness, or tenderness

☐ Swelling of fingers or toes

☐ Fatigue

☐ Morning stiffness

☐ Eye problems

☐ Difficulty dressing

☐ Difficulty walking

11. Please indicate which of the following symptoms of psoriatic arthritis your patients with psoriasis and psoriatic arthritis report least often.
(*Please check only one.*)

- ☐ Joint pain, soreness, or tenderness [If not selected in Q9]
- ☐ Swelling of fingers or toes [If not selected in Q9]
- ☐ Fatigue [If not selected in Q9]
- ☐ Morning stiffness [If not selected in Q9]
- ☐ Eye problems [If not selected in Q9]
- ☐ Difficulty dressing [If not selected in Q9]
- ☐ Difficulty walking [If not selected in Q9]

Impact of Psoriasis and Psoriatic Arthritis on Daily Activities

Impact on Daily Activities	Description
Difficulty with work or school activities	Difficulty doing normal work or schoolwork because of psoriasis or psoriatic arthritis
Difficulty with social or leisure activities	Difficulty doing normal social or leisure activities because of psoriasis or psoriatic arthritis
Difficulty going shopping or doing housework or yard work	Difficulty going shopping or looking after the home or yard because of psoriasis or psoriatic arthritis
Difficulty sleeping	Having poor sleep quality or sleep interruptions because of psoriasis or psoriatic arthritis
Discomfort while doing everyday tasks	Discomfort doing everyday tasks because of psoriasis or psoriatic arthritis
Problems with relationships	Problems with partner, close friends, or family because of psoriasis or psoriatic arthritis

12. Please indicate which of the following impacts on daily activities your patients with psoriasis or psoriatic arthritis report most often. *(Please check only one.)*

- ☐ Difficulty with work or school activities
- ☐ Difficulty with social or leisure activities
- ☐ Difficulty going shopping or doing housework or yard work
- ☐ Difficulty sleeping
- ☐ Discomfort while doing everyday tasks
- ☐ Problems with relationships

13. Please indicate which of the following impacts on daily activities your patients with psoriasis or psoriatic arthritis report least often. *(Please check only one.)*

- ☐ Difficulty with work or school activities [If not selected in Q11]
- ☐ Difficulty with social or leisure activities [If not selected in Q11
- ☐ Difficulty going shopping or doing housework [If not selected in Q11]
- ☐ Difficulty sleeping [If not selected in Q11]
- ☐ Discomfort while doing everyday tasks [If not selected in Q11]
- ☐ Problems with relationships [If not selected in Q11]

Burden of Symptoms of Psoriasis and Psoriatic Arthritis

In the previous questions, we asked you how often your patients indicate that they are affected by different symptoms and impacts on daily activities of psoriasis and psoriatic arthritis.

For the remainder of this survey, we will ask you to think about how burdensome these symptoms and impacts on daily activities are to patients with psoriasis or psoriatic arthritis.

THIS PAGE IS FOR PROGRAMMING PURPOSES ONLY

The following variables will be referenced in the survey.

**Variable**	***gender***	***severity_1***	***severity_2***	***bsa_1***	***bsa_2***	***joints_1***	***joints_2***
**Assignment Method**	randomly assigned	randomly assigned	randomly assigned but cannot equal severity_1	determined by severity_1	determined by severity_2	determined by severity_1	determined by severity_2
**Variable values**	***female*** or ***male***	***mild,*** ***moderate,*** or ***severe***	***mild,*** ***moderate,*** or ***severe***	if severity_1= mild: ***2%***	if severity_2= mild: ***2%***	if severity_1= mild: ***4***	if severity_2= mild: ***4***
				if severity_1= moderate: ***7%***	if severity_2= moderate: ***7%***	if severity_1= moderate: ***8***	if severity_2= moderate: ***8***
				if severity_1= severe: ***15%***	if severity_2= severe: ***15%***	if severity_1= severe: ***11***	if severity_2= severe: ***11***

We are interested in knowing how burdensome you think each of the symptoms and impacts on daily activities described earlier is to patients with psoriasis or psoriatic arthritis.

In the following questions, we will ask you to think about 8 hypothetical patients.

- Each of these patients is [gender].
- Each of these patients has plaques of [severity_1] severity covering [bsa_1] body surface area (BSA).
- Each of these patients has [joints_1] painful, swollen, or tender joints.

Suppose that a [gender] patient has [severity_1] plaques covering [bsa_1] BSA and [joints_1] affected joints and reports [<he/she>] is experiencing the 5 symptoms or impacts on daily activities listed below.

In your opinion, please tell us which one is <u>most bothersome for the patient</u>. In other words, which symptom or impact do you think the patient will want <u>to avoid the most</u>?

[Questions for Patient 1 – 8 are the same as those for Patient 9 – 16. Each respondent will be randomly assigned to one of two blocks of eight questions. See spreadsheet for the symptoms that populate the questions.]

Patient 1

MOST bothersome (Check only ONE)	Symptom
☐	
☐	
☐	
☐	
☐	

[Do not allow respondents to select the feature checked in the previous question]

Now, please tell us which of these same 5 symptoms or impacts you think bothers this patient the least. In other words, which symptom or impact do you think the patient will choose if she has to experience one of these symptoms?

Symptom	LEAST bothersome (Check only ONE)
	☐
	☐
	☐
	☐
	☐

In the next 7 questions, we will show you different sets of 5 symptoms or impacts. For each set of symptoms or impacts, suppose a patient reports [<he/she>] is experiencing these 5 symptoms or impacts.

Please select the symptom or impact you think is most bothersome to the patient by checking the box to the left of the symptom or impact.

Then, please select the symptom or impact that is least bothersome to the patient by checking the box to the right of that symptom or impact.

Remember:

- Each of these patients is [gender].
- Each of these patients has plaques of [severity_1] severity covering [bsa_1] body surface area (BSA).
- Each of these patients has [joints_1] painful, swollen, or tender joints.

In each question, please choose only 1 symptom or impact as the most bothersome and 1 symptom or impact as the least bothersome.

Patient 2

Suppose this patient ([gender] with [severity_1] plaques, [bsa_1] BSA, and [joints_1] affected joints) tells you [<he/she>] is experiencing the 5 symptoms in the list below. In your opinion, which of these symptoms is the most bothersome and least bothersome to this patient?

MOST bothersome (Check only ONE)	Symptom	LEAST bothersome (Check only ONE)
☐		☐
☐		☐
☐		☐
☐		☐
☐		☐

Patient 3

Suppose this patient ([gender] with [severity_1] plaques, [bsa_1] BSA, and [joints_1] affected joints) tells you [<he/she>] is experiencing the 5 symptoms in the list below. In your opinion, which of these symptoms is the most bothersome and least bothersome to this patient?

MOST bothersome (Check only ONE)	Symptom	LEAST bothersome (Check only ONE)
☐		☐
☐		☐
☐		☐
☐		☐
☐		☐

Patient 4

Suppose this patient ([gender] with [severity_1] plaques, [bsa_1] BSA, and [joints_1] affected joints) tells you [<he/she>] is experiencing the 5 symptoms in the list below. In your opinion, which of these symptoms is the most bothersome and least bothersome to this patient?

MOST bothersome (Check only ONE)	Symptom	LEAST bothersome (Check only ONE)
☐		☐
☐		☐
☐		☐
☐		☐
☐		☐

Patient 5

Suppose this patient ([gender] with [severity_1] plaques, [bsa_1] BSA, and [joints_1] affected joints) tells you [<he/she>] is experiencing the 5 symptoms in the list below. In your opinion, which of these symptoms is the most bothersome and least bothersome to this patient?

MOST bothersome (Check only ONE)	Symptom	LEAST bothersome (Check only ONE)
☐		☐
☐		☐
☐		☐
☐		☐
☐		☐

Patient 6

Suppose this patient ([gender] with [severity_1] plaques, [bsa_1] BSA, and [joints_1] affected joints) tells you [<he/she>] is experiencing the 5 symptoms in the list below. In your opinion, which of these symptoms is the most bothersome and least bothersome to this patient?

MOST bothersome (Check only ONE)	Symptom	LEAST bothersome (Check only ONE)
☐		☐
☐		☐
☐		☐
☐		☐
☐		☐

Patient 7

Suppose this patient ([gender] with [severity_1] plaques, [bsa_1] BSA, and [joints_1] affected joints) tells you [<he/she>] is experiencing the 5 symptoms in the list below. In your opinion, which of these symptoms is the most bothersome and least bothersome to this patient?

MOST bothersome (Check only ONE)	Symptom	LEAST bothersome (Check only ONE)
☐		☐
☐		☐
☐		☐
☐		☐
☐		☐

Patient 8

Suppose this patient ([gender] with [severity_1] plaques, [bsa_1] BSA, and [joints_1] affected joints) tells you [<he/she>] is experiencing the 5 symptoms in the list below. In your opinion, which of these symptoms is the most bothersome and least bothersome to this patient?

MOST bothersome (Check only ONE)	Symptom	LEAST bothersome (Check only ONE)
☐		☐
☐		☐
☐		☐
☐		☐
☐		☐

In the following questions, we will ask you to think about 8 additional hypothetical patients.

Remember:

- Each of these patients is [gender].
- Each of these patients has plaques of [severity_2] severity covering [bsa_2] body surface area (BSA).
- Each of these patients has [joints_2] painful, swollen, or tender joints.

Patient 9

Suppose this patient ([gender] with [severity_2] plaques, [bsa_2] BSA, and [joints_2] affected joints) tells you [<he/she>] is experiencing the 5 symptoms in the list below. In your opinion, which of these symptoms is the most bothersome and least bothersome to this patient?

MOST bothersome (Check only ONE)	Symptom	LEAST bothersome (Check only ONE)
☐		☐
☐		☐
☐		☐
☐		☐
☐		☐

Patient 10

Suppose this patient ([gender] with [severity_2] plaques, [bsa_2] BSA, and [joints_2] affected joints) tells you [<he/she>] is experiencing the 5 symptoms in the list below. In your opinion, which of these symptoms is the most bothersome and least bothersome to this patient?

MOST bothersome (Check only ONE)	Symptom	LEAST bothersome (Check only ONE)
☐		☐
☐		☐
☐		☐
☐		☐
☐		☐

Patient 11

Suppose this patient ([gender] with [severity_2] plaques, [bsa_2] BSA, and [joints_2] affected joints) tells you [<he/she>] is experiencing the 5 symptoms in the list below. In your opinion, which of these symptoms is the most bothersome and least bothersome to this patient?

MOST bothersome (Check only ONE)	Symptom	LEAST bothersome (Check only ONE)
☐		☐
☐		☐
☐		☐
☐		☐
☐		☐

Patient 12

Suppose this patient ([gender] with [severity_2] plaques, [bsa_2] BSA, and [joints_2] affected joints) tells you [<he/she>] is experiencing the 5 symptoms in the list below. In your opinion, which of these symptoms is the most bothersome and least bothersome to this patient?

MOST bothersome (Check only ONE)	Symptom	LEAST bothersome (Check only ONE)
☐		☐
☐		☐
☐		☐
☐		☐
☐		☐

Patient 13

Suppose this patient ([gender] with [severity_2] plaques, [bsa_2] BSA, and [joints_2] affected joints) tells you [<he/she>] is experiencing the 5 symptoms in the list below. In your opinion, which of these symptoms is the most bothersome and least bothersome to this patient?

MOST bothersome (Check only ONE)	Symptom	LEAST bothersome (Check only ONE)
☐		☐
☐		☐
☐		☐
☐		☐
☐		☐

Patient 14

Suppose this patient ([gender] with [severity_2] plaques, [bsa_2] BSA, and [joints_2] affected joints) tells you [<he/she>] is experiencing the 5 symptoms in the list below. In your opinion, which of these symptoms is the most bothersome and least bothersome to this patient?

MOST bothersome (Check only ONE)	Symptom	LEAST bothersome (Check only ONE)
☐		☐
☐		☐
☐		☐
☐		☐
☐		☐

Patient 15

Suppose this patient ([gender] with [severity_2] plaques, [bsa_2] BSA, and [joints_2] affected joints) tells you [<he/she>] is experiencing the 5 symptoms in the list below. In your opinion, which of these symptoms is the most bothersome and least bothersome to this patient?

MOST bothersome (Check only ONE)	Symptom	LEAST bothersome (Check only ONE)
☐		☐
☐		☐
☐		☐
☐		☐
☐		☐

Patient 16

Suppose this patient ([gender] with [severity_2] plaques, [bsa_2] BSA, and [joints_2] affected joints) tells you [<he/she>] is experiencing the 5 symptoms in the list below. In your opinion, which of these symptoms is the most bothersome and least bothersome to this patient?

MOST bothersome (Check only ONE)	Symptom	LEAST bothersome (Check only ONE)
☐		☐
☐		☐
☐		☐
☐		☐
☐		☐

Other Questions About You

14. In what year were you born? ___________

15. What is your gender?

☐ Female

☐ Male

☐ Prefer not to answer

16. In which state or states are you licensed to practice?

____
