# Supplementary material for: Comparison of US patient, rheumatologist, and dermatologist perceptions of psoriatic disease symptoms: results from the DISCONNECT study
Source: Arthritis Res Ther. 2018 May 31;20:102. doi: 10.1186/s13075-018-1601-4 (PMC5977464; doi:10.1186/s13075-018-1601-4)
Supplement: Supplementary file 3 — Results of patient subgroup analyses (DOCX 1800 kb) [file 13075_2018_1601_MOESM3_ESM.docx]

# Additional File 3: Subgroup Analyses

1. Overview of Patient Subgroup Analysis (N = 200)

| **Patient Subgroup Pair** | **Patient Subgroup** | **n (%)** | **Statistically Significant Difference in All Reponses (*P* < 0.05) Overall?^a^** |
| --- | --- | --- | --- |
| Age | Above median age of 37.5 years  (Adjustment group) | 100 (50.0%) | Yes, *P* < 0.01 |
|  | Equal to or below median age of 37.5 years  (Baseline group) | 100 (50.0%) |  |
| Gender | Female  (Adjustment group) | 101 (50.5%) | Yes, *P* < 0.01 |
|  | Male  (Baseline group) | 99 (49.5%) |  |
| Severity of skin symptoms | Above median of 7 for Q17 – rating of severity of skin symptoms in the past week  (Adjustment group) | 92 (46.0%) | Yes, *P* < 0.01 |
|  | Equal to or below median of 7 for Q17 – rating of severity of skin symptoms in the past week  (Baseline group) | 108 (54.0%) |  |
| Severity of joint symptoms | Above median of 7 for Q21 – rating of severity of joint symptoms in the past week  (Adjustment group) | 73 (36.5%) | Yes, *P* < 0.01 |
|  | Equal to or below median of 7 for Q21 – rating of severity of joint symptoms in the past week  (Baseline group) | 127 (63.5%) |  |
| BSA | 6 or greater hand areas of BSA  (Adjustment group) | 88 (44.0%) | Yes, *P* = 0.032 |
|  | 5 or fewer hand areas of BSA  (Baseline group) | 112 (56.0%) |  |
| Relationship status | Married/living as married/civil partnership  (Adjustment group) | 142 (71.0%) | Yes, *P* < 0.01 |
|  | Not Married/living as married/civil partnership  (Baseline group) | 58 (29.0%) |  |
| Psoriasis on face and/or neck | Psoriasis patches on face and/or neck in the past week  (Adjustment group) | 100 (50.0%) | Yes, *P* < 0.01 |
|  | No psoriasis patches on face and/or neck in the past week | 100 (50.0%) |  |
| Time since diagnosis | Psoriasis and/or psoriatic arthritis for at least 2 years  (Adjustment group) | 107 (53.5%) | Yes, *P* < 0.01 |
|  | Psoriasis and/or psoriatic arthritis for less than 2 years  (Baseline group) | 93 (46.5%) |  |
| Painful skin | Experienced painful skin in the past week  (Adjustment group) | 63 (31.5%) | Yes, *P* < 0.01 |
|  | Did not experience painful skin in the past week  (Baseline group) | 137 (68.5%) |  |
| Currently taking injectable or IV treatment | Currently taking injectable or IV treatment  (Adjustment group) | 62 (31.0%) | Yes, *P* < 0.01 |
|  | Not currently taking injectable or IV treatment  (Baseline group) | 138 (69.0%) |  |
| Currently taking oral, injectable, or IV treatment | Currently taking oral, injectable, or IV treatment  (Adjustment group) | 111 (55.5%) | Yes, *P* < 0.01 |
|  | Not currently taking oral, injectable, or IV treatment  (Baseline group) | 89 (44.5%) |  |

BSA = body surface area; IV = intravenous.

^a^ Statistical significance was estimated based on random-parameters logit models with 100 draws in NLOGIT.

1. Best-Worst Scaling Relative-Bother Estimates: Patient Age Subgroups (N = 200)


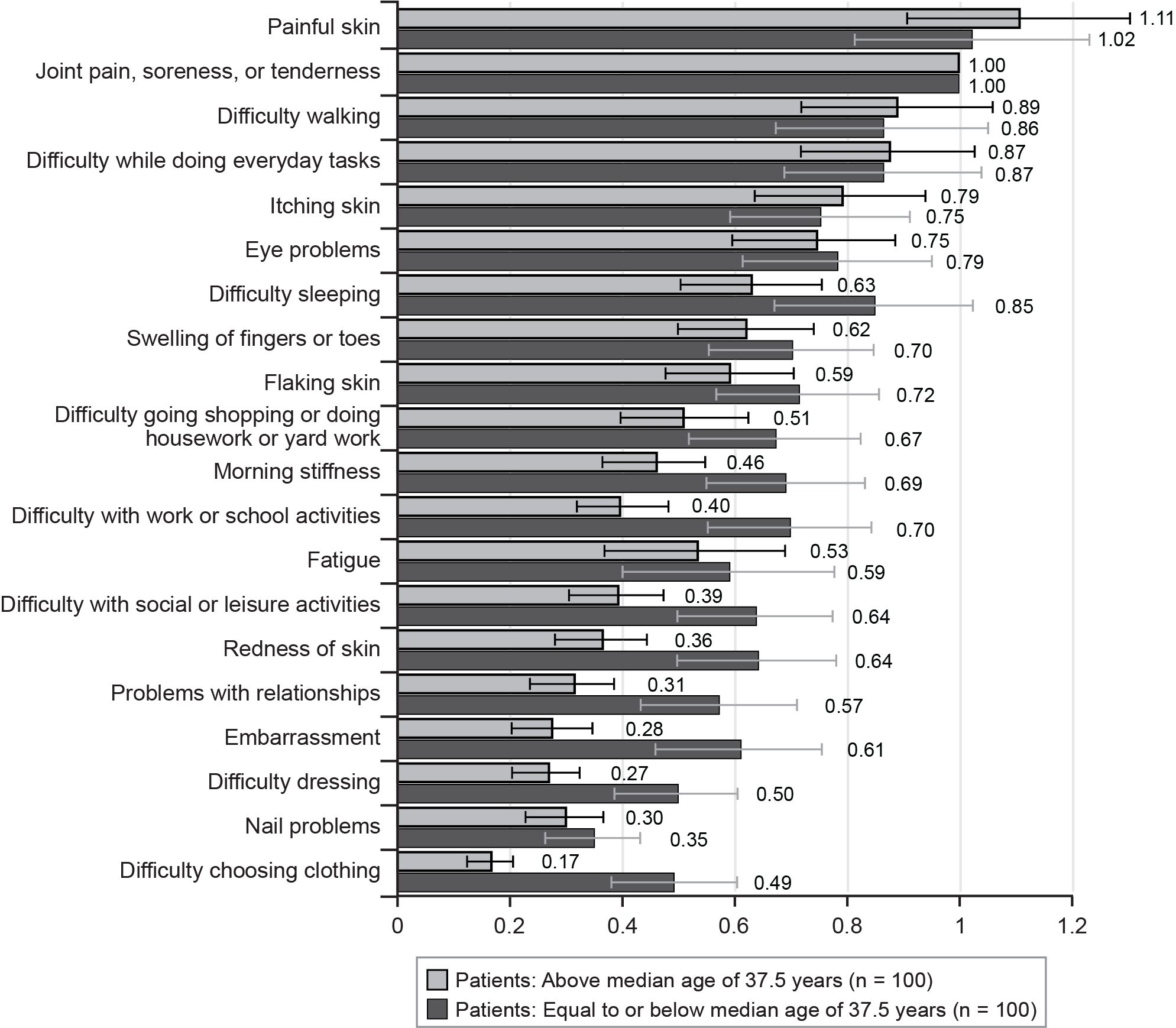


Note: The bars surrounding each mean importance weight denote the 95% confidence interval about the point estimate.

1. Best-Worst Scaling Relative-Bother Estimates: Patient Gender Subgroups (N = 200)


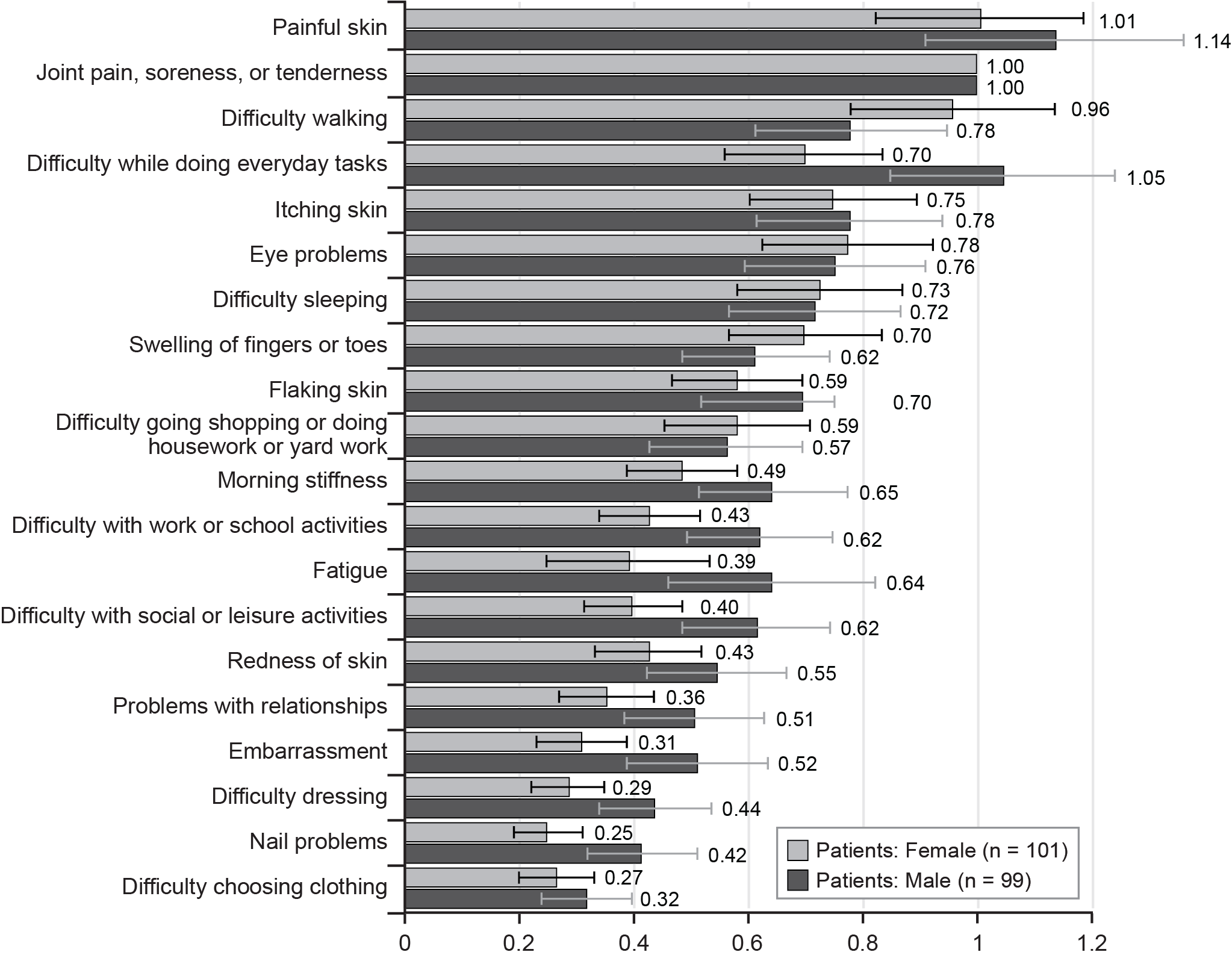


Note: The bars surrounding each mean importance weight denote the 95% confidence interval about the point estimate.

1. Best-Worst Scaling Relative-Bother Estimates: Skin Severity Patient Subgroups (N = 200)


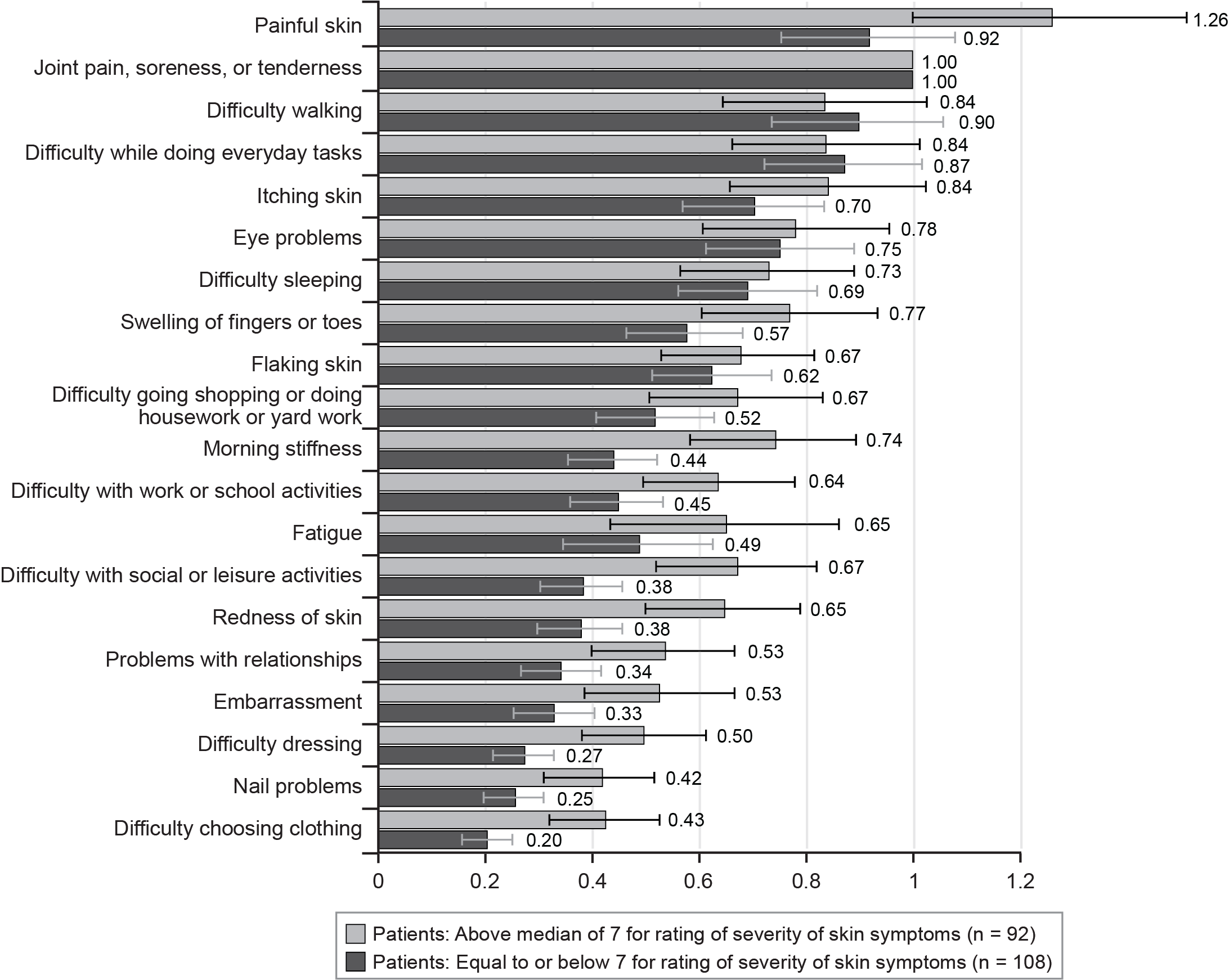


Note: The bars surrounding each mean importance weight denote the 95% confidence interval about the point estimate.

1. Best-Worst Scaling Relative-Bother Estimates: Joint Severity Patient Subgroups (N = 200)


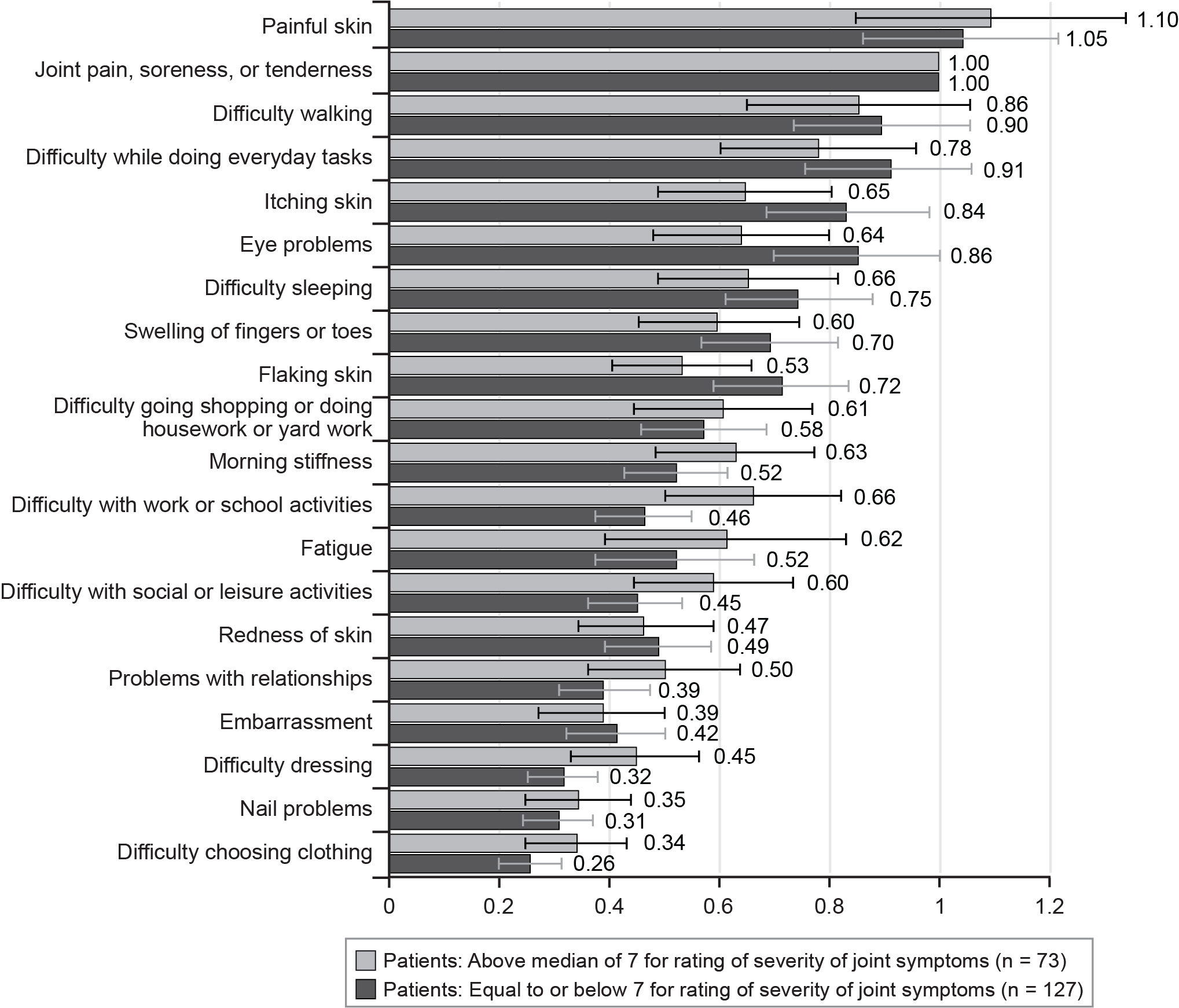


Note: The bars surrounding each mean importance weight denote the 95% confidence interval about the point estimate.

1. Best-Worst Scaling Relative-Bother Estimates: Body Surface Area Patient Subgroups (N = 200)


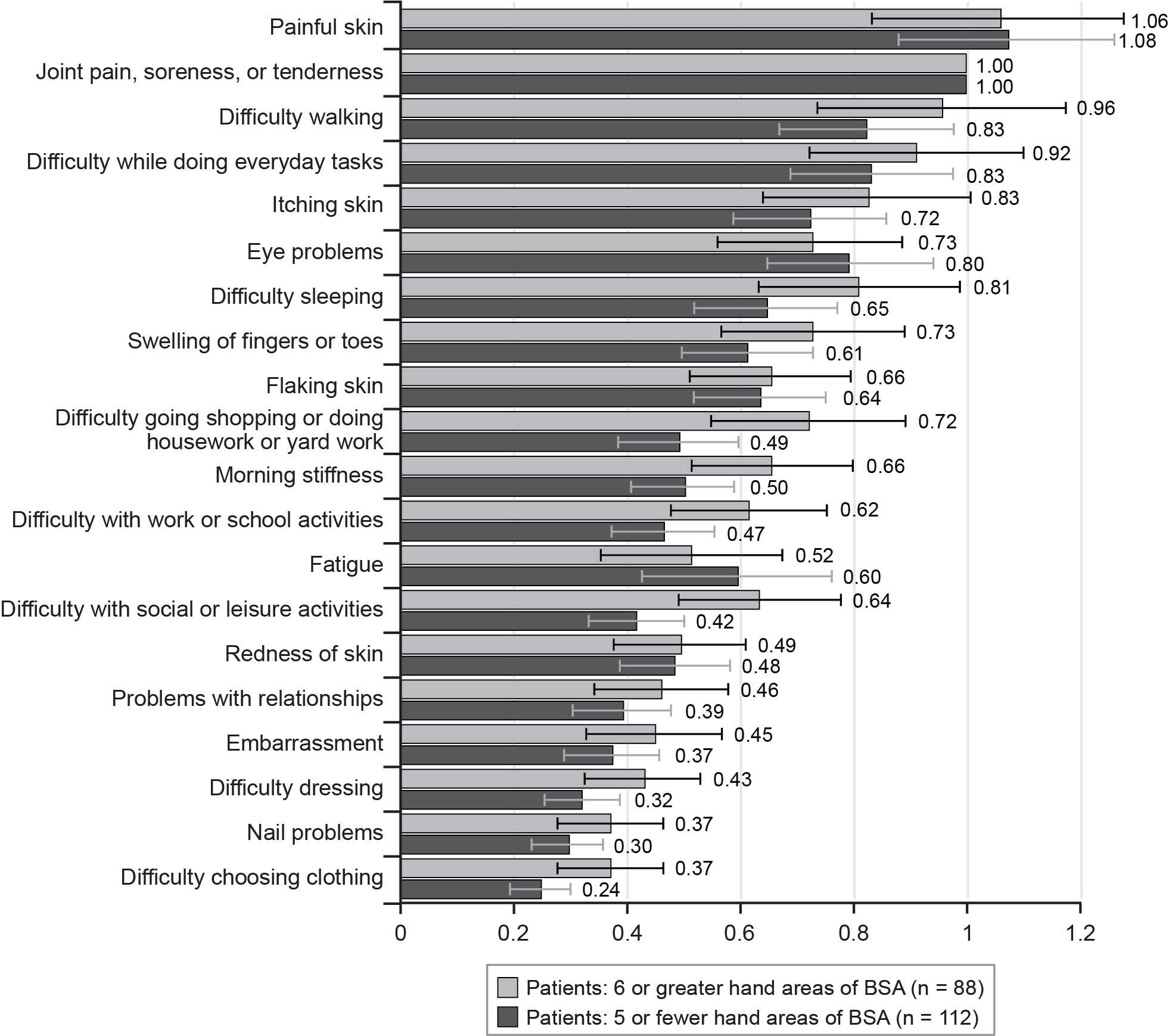


BSA = body surface area.

Note: The bars surrounding each mean importance weight denote the 95% confidence interval about the point estimate.

1. Best-Worst Scaling Relative-Bother Estimates: Patient Relationship Status Subgroups (N = 200)


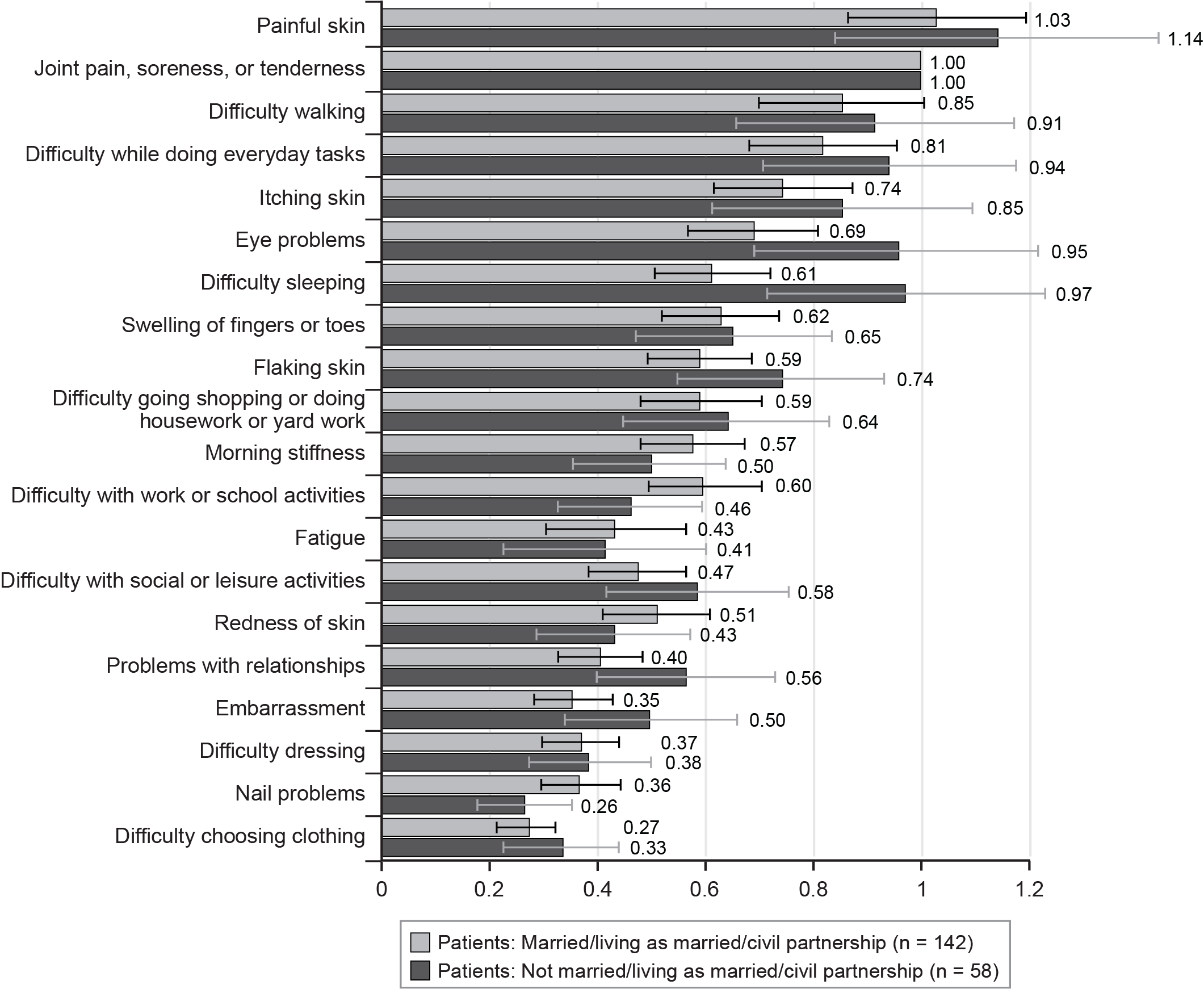


Note: The bars surrounding each mean importance weight denote the 95% confidence interval about the point estimate.

1. Best-Worst Scaling Relative-Bother Estimates: Psoriasis on Face and/or Neck Subgroups (N = 200)


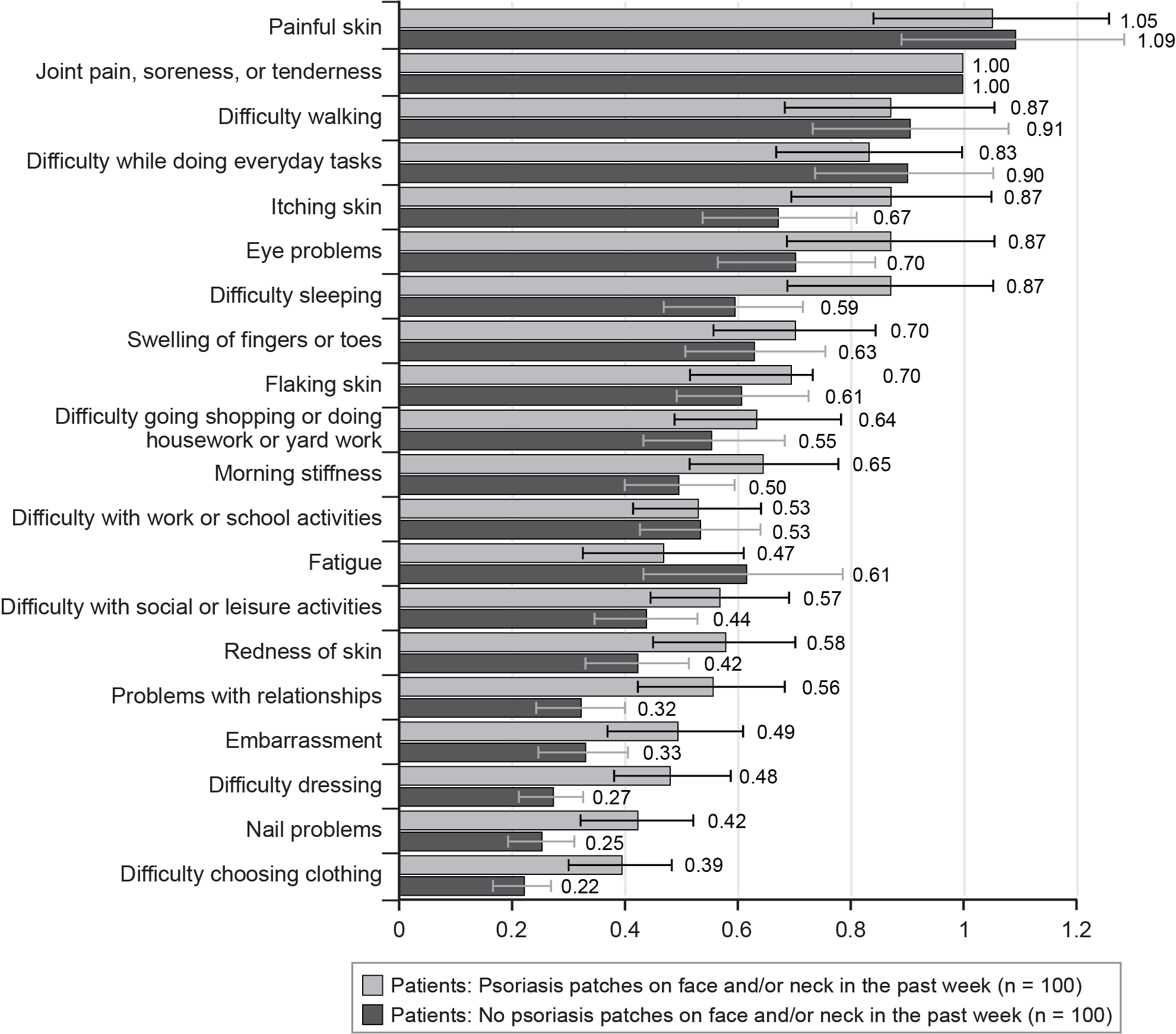


Note: The bars surrounding each mean importance weight denote the 95% confidence interval about the point estimate.

1. Best-Worst Scaling Relative-Bother Estimates: Time Since Diagnosis Subgroups (N = 200)


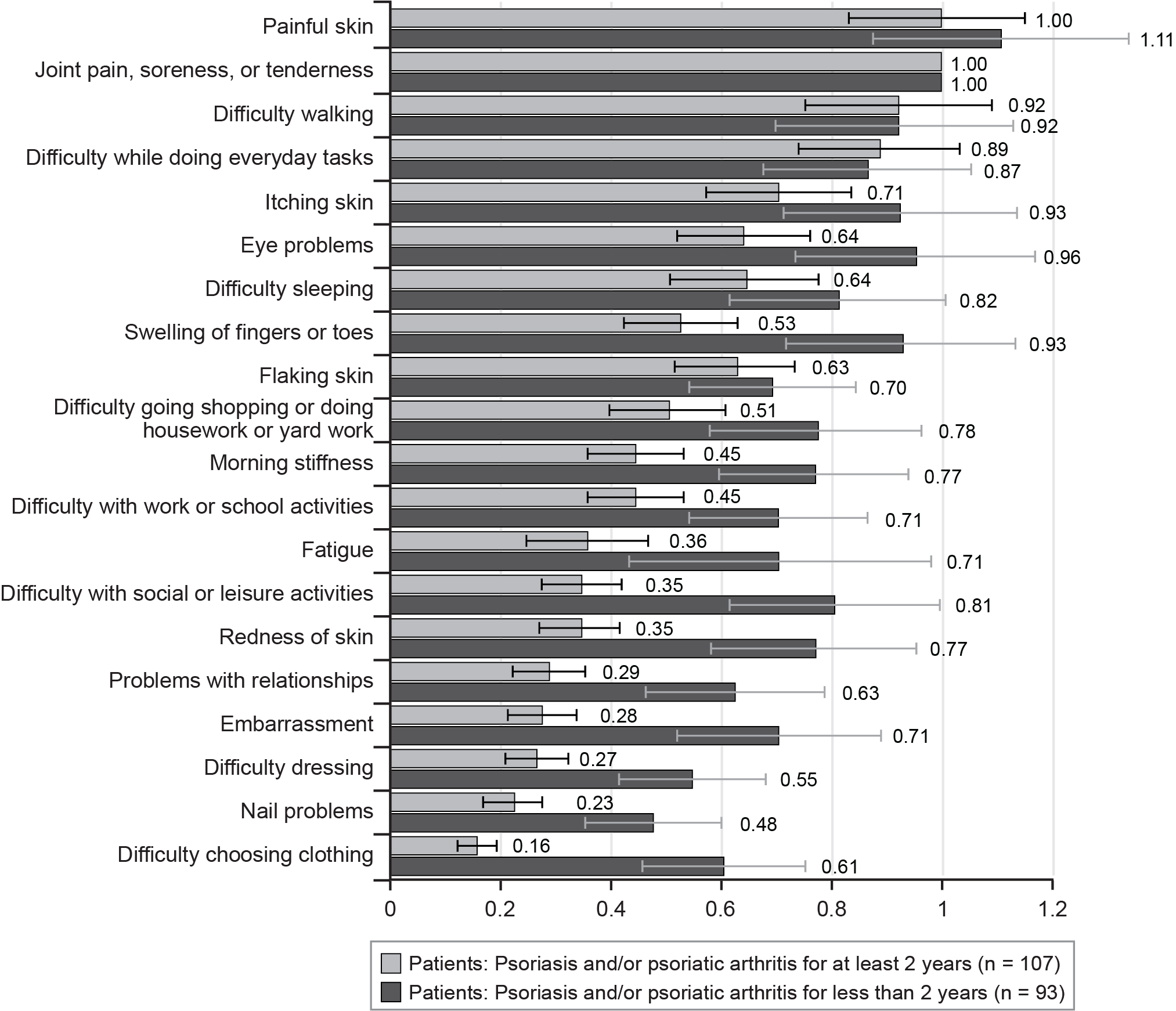


Note: The bars surrounding each mean importance weight denote the 95% confidence interval about the point estimate.

1. Best-Worst Scaling Relative-Bother Estimates: Painful Skin Subgroups (N = 200)


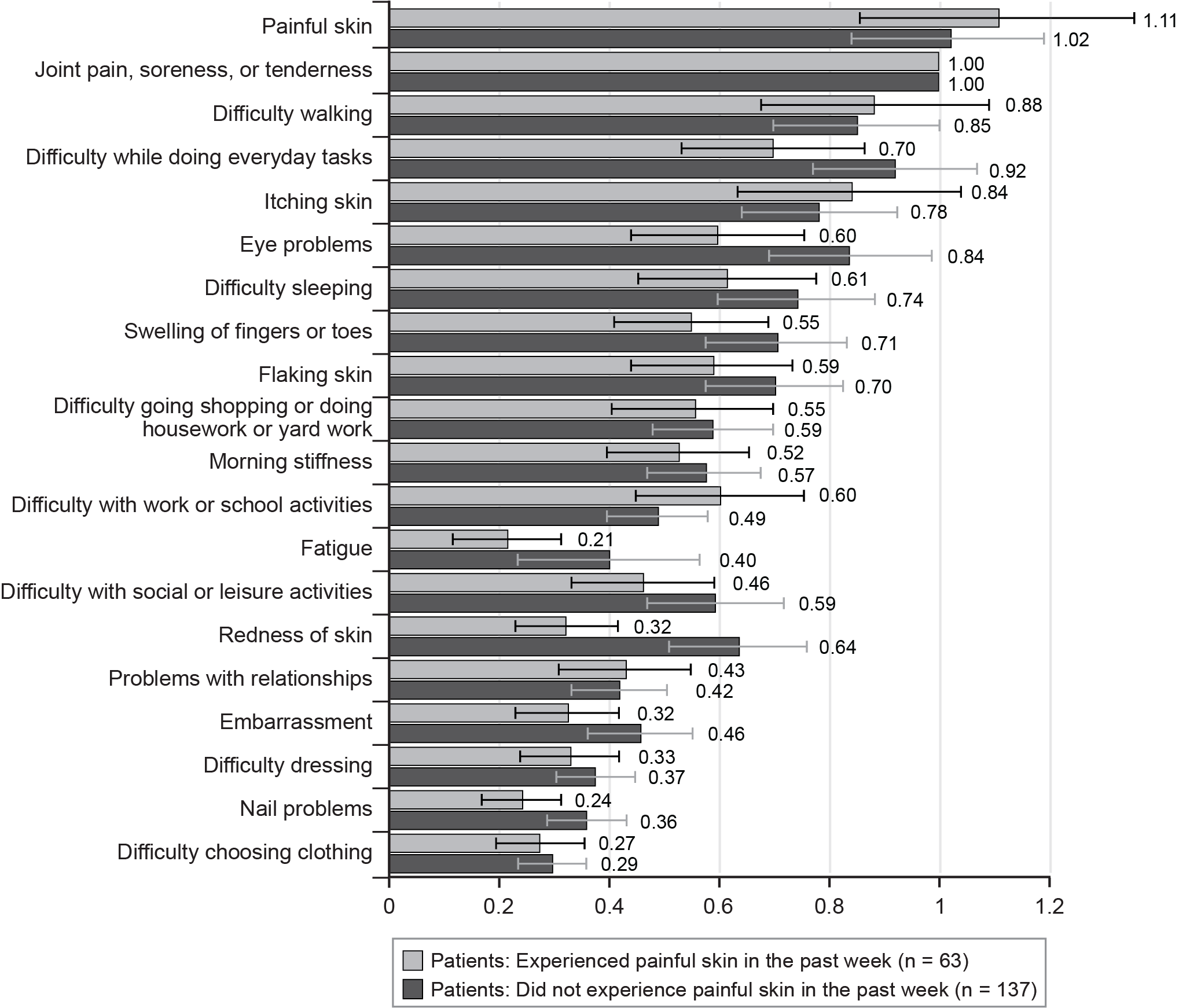


Note: The bars surrounding each mean importance weight denote the 95% confidence interval about the point estimate.

1. Best-Worst Scaling Relative-Bother Estimates: Current Treatment (Injectable or IV vs. Others) Subgroups (N = 200)


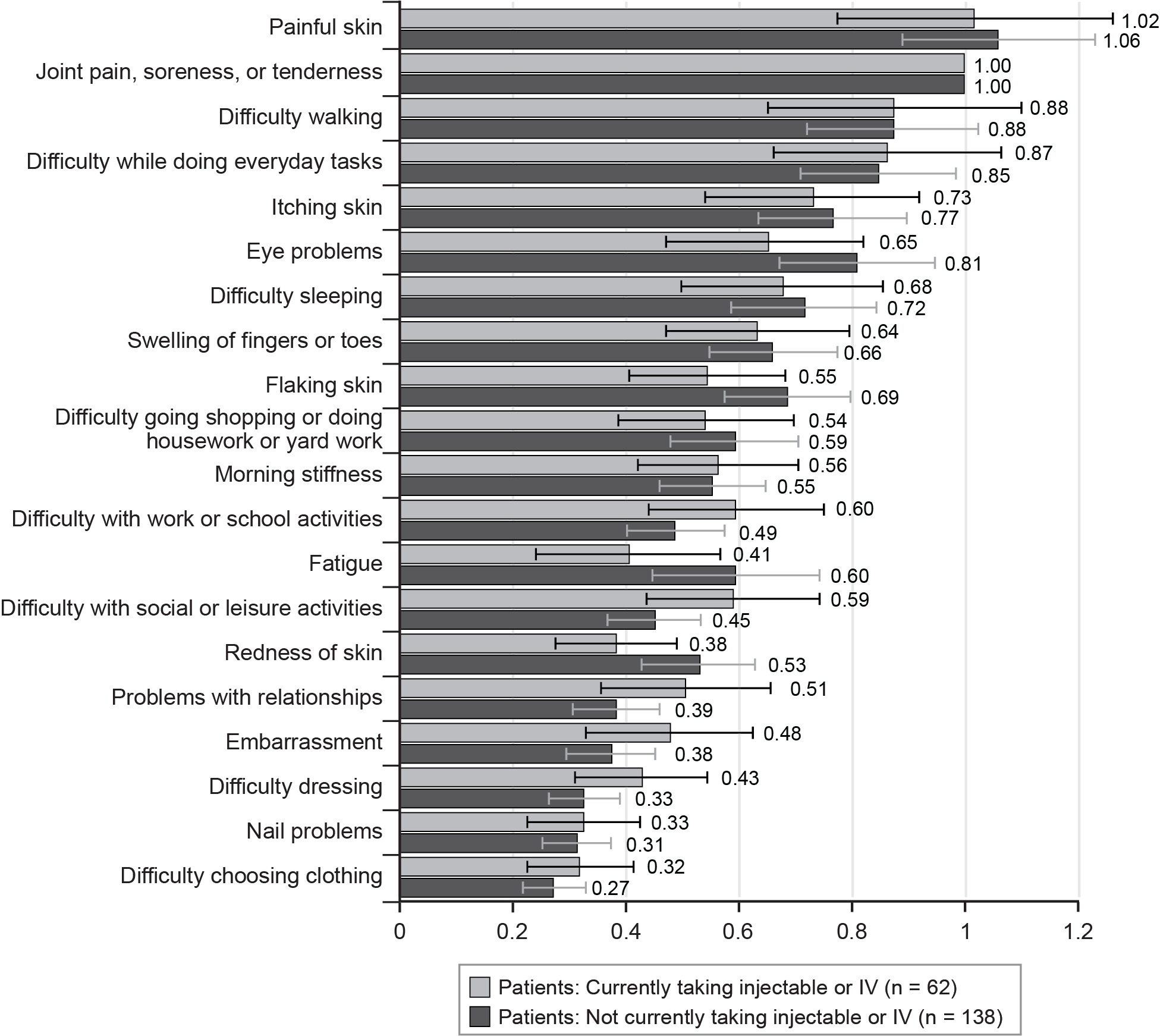


IV = intravenous.

Note: The bars surrounding each mean importance weight denote the 95% confidence interval about the point estimate.

1. Best-Worst Scaling Relative-Bother Estimates: Current Treatment (Injectable, IV, or Oral vs. Others) Subgroups (N = 200)


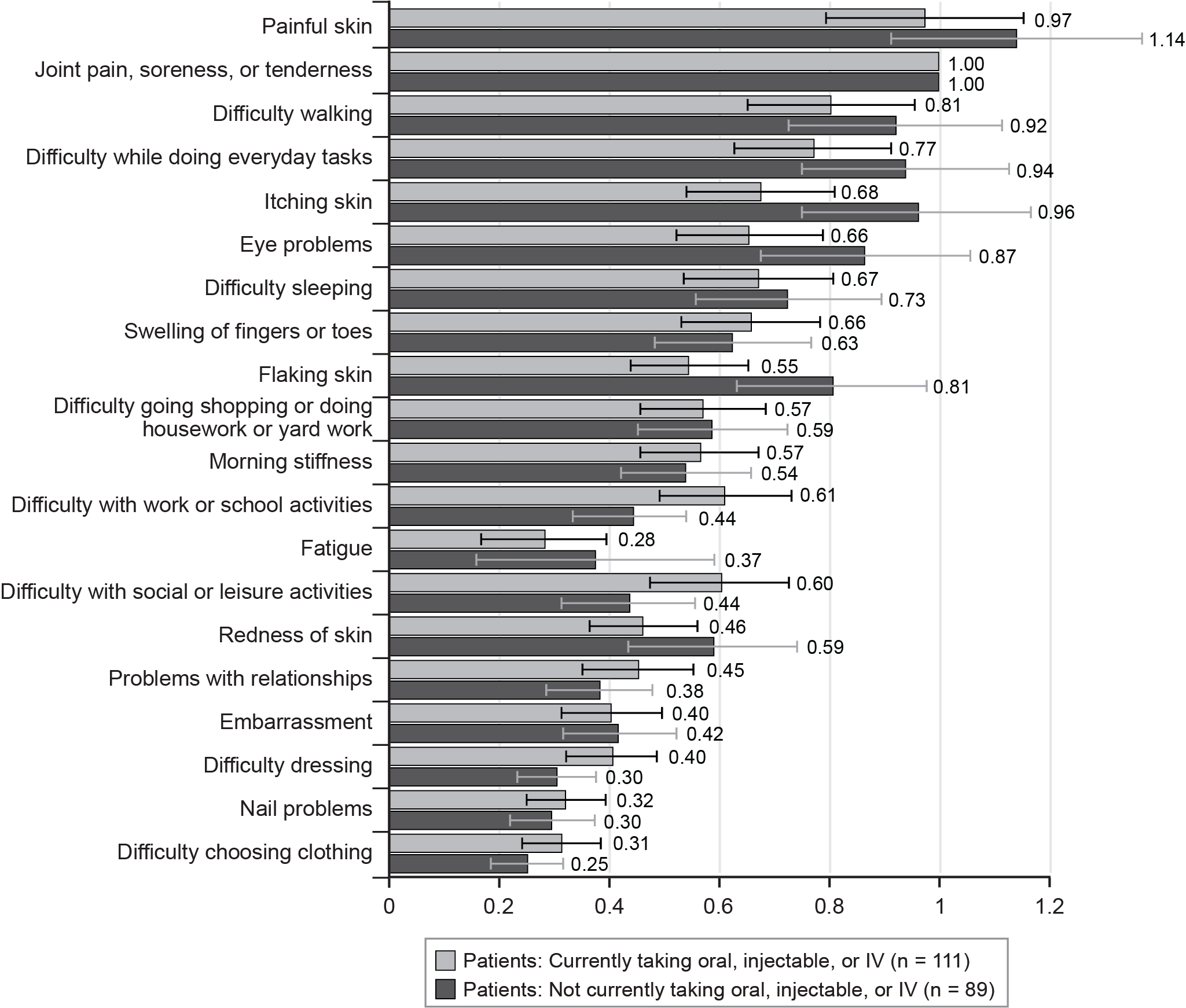


IV = intravenous.

Note: The bars surrounding each mean importance weight denote the 95% confidence interval about the point estimate.
