# Supplementary material for: Comparison of US patient, rheumatologist, and dermatologist perceptions of psoriatic disease symptoms: results from the DISCONNECT study
Source: Arthritis Res Ther. 2018 May 31;20:102. doi: 10.1186/s13075-018-1601-4 (PMC5977464; doi:10.1186/s13075-018-1601-4)
Supplement: Supplementary file 4 — Patients’ experience with survey items and physicians’ medical experience and experience with survey items. (DOCX 45 kb) [file 13075_2018_1601_MOESM4_ESM.docx]

# Additional File 4: Patients’ experience with survey items and Physicians’ Medical Experience and experience with survey items

Table D-1 presents details of the percentages of patients with experience ever or in the last week with each of the 20 items included in the survey. Of the seven skin symptoms included as survey items, 81% of patients reported having ever experienced itching skin, 75% redness of skin, 70% flaking skin, and 56% painful skin. Of the seven joint symptoms included as survey items, 67% of patients reported having ever experienced joint pain, soreness, or tenderness; 51% fatigue; 53% morning stiffness; and 48% swelling of fingers or toes. Of the six impacts on daily activities included as survey items, 53% of patients reported having had experienced difficulty with social or leisure activities, 47% difficulty sleeping, 46% difficulty while doing everyday tasks, and 40% difficulty going shopping or doing housework or yard work. For all of the items included in the survey, the mean severity was rated to be between 6 and 8 on a scale from 0 to 10, indicating a moderate level of severity. Pearson’s chi-squared test showed no difference between rheumatologists and dermatologists (at the 5% level) in their responses for skin and joint symptoms but a statistically significant difference for reporting of the impact of their symptoms on daily activities, with 59% of the rheumatologists responding that the most commonly reported impact on daily activity was discomfort while doing everyday tasks (59%) compared with 33% of the dermatologists. The percentages for the most and least reported items by patients for the rheumatologists and dermatologists are shown in Table D-3.

1. Patients’ Experience with the Survey Items

| Items | % Ever Experienced | % Experienced in Last Week | Severity in Last Week^a^ |
| --- | --- | --- | --- |
| Skin Symptoms (N = 200)  Itching skin  Redness of skin  Flaking skin  Painful skin  Nail problems  Difficulty choosing clothing  Embarrassment  None in last week | 80.5%  75.0%  69.5%  56.0%  27.0%  34.0%  30.5% | 65.5%  57.5%  51.5%  31.5%  13.5%  18.5%  18.0%  4.0% | 7.2 (2.0)  7.2 (2.0)  7.2 (1.9)  7.7 (1.5)  8.1 (1.9)  7.9 (1.5)  8.3 (1.9) |
| Joint Symptoms (N = 197)  Joint pain, soreness, or tenderness  Swelling of fingers or toes  Fatigue  Morning stiffness  Eye problems  Difficulty dressing  Difficulty walking  None in last week | 66.5%  47.7%  50.8%  52.8%  25.4%  18.3%  19.3% | 54.8%  35.0%  37.6%  35.0%  13.7%  12.7%  15.7%  10.2% | 7.5 (1.9)  7.5 (1.9)  7.6 (2.2)  6.8 (2.2)  7.6 (1.6)  7.9 (1.6)  7.7 (1.8) |
| **Daily Activities (N = 200)**  Difficulty with work or school  Difficulty with social or leisure activities  Difficulty shopping or doing house or yard work  Difficulty sleeping  Discomfort doing everyday tasks  Problems with relationships  None ever or in last week | 38.0%  52.5%  40.0%  46.5%  46.0%  21.0%  9.0% | 33.0%  37.4%  27.5%  38.5%  35.7%  13.2%  7.7% | 7.9 (1.6)  7.4 (1.5)  7.4 (1.9)  7.5 (1.9)  7.0 (2.1)  7.5 (2.1) |

^a^ Mean and standard deviation of severity of those experiencing item in the last week where 0 = none or no difficulty and 10 = extremely severe or extreme difficulty

Table D-2 presents characteristics of the 300 physicians who completed the survey. Physicians were also asked which of the 20 items included in the survey their patients reported most and least often and the responses reported separately for rheumatologists and dermatologists (Table D-3). For skin symptoms, physicians stated that their patients most often reported flaking skin (32%), redness of skin (26%), and itching skin (19%). Physicians stated that their patients least often reported difficulty choosing clothing (48%) and nail problems (20%). Pearson’s chi-squared test showed no difference between dermatologists and rheumatologists (at the 5% level) in these reported frequencies. For joint symptoms, 80% of physicians stated that their patients reported joint pain, soreness, or tenderness most often. Sixty-eight percent of physicians stated that their patients report eye problems least often. Pearson’s chi-squared test showed no difference between dermatologists and rheumatologists (at the 5% level) in these reported frequencies. For daily activities, dermatologists stated that their patients most often reported difficulty with social or leisure activities (35%) and discomfort while doing everyday tasks (33%). More than half of rheumatologists (59%) stated that their patients most often reported discomfort while doing everyday tasks, followed by difficulty with work or school activities (18%). Dermatologists stated that their patients reported difficulty sleeping least often (43%) while rheumatologists stated that their patients reported problems with relationships least often (58%). Pearson’s chi-square test indicates a statistically significant difference between what dermatologists and rheumatologists said their patients reported most and least often from among the six impacts on daily activities.

1. Physicians’ Medical Experience

|  | Statistic or Category | Dermatologists (n = 150) | Rheumatologists (n = 150) | *P* Value | Overall (N = 300) |
| --- | --- | --- | --- | --- | --- |
| All respondents | | | | | |
| How many years have you been in practice since completing your medical training? | n | 150 | 150 |  | 300 |
|  | Less than 1 year | 9 (6.0%) | 4 (2.7%) | 0.006 | 13 (4.3%) |
|  | 1-3 years | 18 (12.0%) | 8 (5.3%) |  | 26 (8.7%) |
|  | 4-6 years | 30 (20.0%) | 18 (12.0%) |  | 48 (16.0%) |
|  | 7-9 years | 18 (12.0%) | 22 (14.7%) |  | 40 (13.3%) |
|  | 10-15 years | 24 (16.0%) | 24 (16.0%) |  | 48 (16.0%) |
|  | 16-20 years | 20 (13.3%) | 16 (10.7%) |  | 36 (12.0%) |
|  | 21-25 years | 14 (9.3%) | 36 (24.0%) |  | 50 (16.7%) |
|  | More than 25 years | 17 (11.3%) | 22 (14.7%) |  | 39 (13.0%) |
|  | Blank/no answer | 0 | 0 |  | 0 |
| Which of the following describes your practice? (Check all that apply.) | n | 150 | 150 |  | 300 |
|  | Office-based private practice | 118 (78.7%) | 114 (76.0%) | 0.581 | 232 (77.3%) |
|  | Hospital-based private practice | 6 (4.0%) | 11 (7.3%) | 0.212 | 17 (5.7%) |
|  | Academic hospital-based practice | 30 (20.0%) | 30 (20.0%) | 1.000 | 60 (20.0%) |
|  | Other | 1 (0.7%) | 2 (1.3%) | 1.000^a^ | 3 (1.0%) |
| On average, how many patients with plaque psoriasis do you treat each month? | n | 150 | 150 |  | 300 |
|  | 5 patients or fewer | 1 (0.7%) | 6 (4.0%) | 0.001 | 7 (2.3%) |
|  | 6-10 patients | 8 (5.3%) | 22 (14.7%) |  | 30 (10.0%) |
|  | 11-20 patients | 17 (11.3%) | 22 (14.7%) |  | 39 (13.0%) |
|  | 21-30 patients | 31 (20.7%) | 29 (19.3%) |  | 60 (20.0%) |
|  | 31-40 patients | 24 (16.0%) | 23 (15.3%) |  | 47 (15.7%) |
|  | 41-50 patients | 14 (9.3%) | 21 (14.0%) |  | 35 (11.7%) |
|  | More than 50 patients | 55 (36.7%) | 27 (18.0%) |  | 82 (27.3%) |
|  | Missing | 1 |  |  | 1 |
| On average, how many patients with psoriatic arthritis do you treat each month? | n | 150 | 150 |  | 300 |
|  | 5 patients or fewer | 30 (20.0%) | 0 | 0.000 | 30 (10.0%) |
|  | 6-10 patients | 38 (25.3%) | 14 (9.3%) |  | 52 (17.3%) |
|  | 11-20 patients | 34 (22.7%) | 19 (12.7%) |  | 53 (17.7%) |
|  | 21-30 patients | 21 (14.0%) | 23 (15.3%) |  | 44 (14.7%) |
|  | 31-40 patients | 11 (7.3%) | 27 (18.0%) |  | 38 (12.7%) |
|  | 41-50 patients | 7 (4.7%) | 28 (18.7%) |  | 35 (11.7%) |
|  | More than 50 patients | 9 (6.0%) | 39 (26.0%) |  | 48 (16.0%) |
| For what percentage of your patients do you prescribe biologic agents to treat psoriasis or psoriatic arthritis? | n | 150 | 150 |  | 300 |
|  | I do not prescribe biologic agents | 1 (0.7%) | 0 | 0.000 | 1 (0.3%) |
|  | More than 0%, but less than 25% | 19 (12.7%) | 5 (3.3%) |  | 24 (8.0%) |
|  | 26%-50% | 76 (50.7%) | 30 (20.0%) |  | 106 (35.3%) |
|  | 51%-75% | 39 (26.0%) | 85 (56.7%) |  | 124 (41.3%) |
|  | 76%-100% | 15 (10.0%) | 30 (20.0%) |  | 45 (15.0%) |
| Among respondents who prescribe biologic agents to treat psoriasis or psoriatic arthritis | | | | | |
| How long have you been prescribing biologic agents to treat patients with psoriasis or psoriatic arthritis? | n | 149 | 150 |  | 299 |
|  | Less than 1 year | 6 (4.0%) | 1 (0.7%) | 0.000 | 7 (2.3%) |
|  | 1-2 years | 12 (8.1%) | 4 (2.7%) |  | 16 (5.4%) |
|  | 3-5 years | 46 (30.9%) | 21 (14.0%) |  | 67 (22.4%) |
|  | More than 5 years | 85 (57.0%) | 124 (82.7%) |  | 209 (69.9%) |
| All respondents | | | | | |
| Please indicate which of the following symptoms of psoriasis your patients with psoriasis or psoriatic arthritis report most often. (Please check only one.) | n | 150 | 150 |  | 300 |
|  | Itching skin | 32 (21.3%) | 25 (16.7%) | 0.052 | 57 (19.0%) |
|  | Redness of skin | 37 (24.7%) | 40 (26.7%) |  | 77 (25.7%) |
|  | Flaking skin | 57 (38.0%) | 40 (26.7%) |  | 97 (32.3%) |
|  | Painful skin | 7 (4.7%) | 14 (9.3%) |  | 21 (7.0%) |
|  | Nail problems | 1 (0.7%) | 6 (4.0%) |  | 7 (2.3%) |
|  | Difficulty choosing clothing | 0 | 1 (0.7%) |  | 1 (0.3%) |
|  | Embarrassment | 16 (10.7%) | 24 (16.0%) |  | 40 (13.3%) |
| Please indicate which of the following symptoms of psoriasis your patients with psoriasis or psoriatic arthritis report least often. | n | 150 | 150 |  | 300 |
|  | Itching skin | 7 (4.7%) | 8 (5.3%) | 0.956 | 15 (5.0%) |
|  | Redness of skin | 4 (2.7%) | 6 (4.0%) |  | 10 (3.3%) |
|  | Flaking skin | 3 (2.0%) | 3 (2.0%) |  | 6 (2.0%) |
|  | Painful skin | 27 (18.0%) | 25 (16.7%) |  | 52 (17.3%) |
|  | Nail problems | 29 (19.3%) | 31 (20.7%) |  | 60 (20.0%) |
|  | Difficulty choosing clothing | 75 (50.0%) | 69 (46.0%) |  | 144 (48.0%) |
|  | Embarrassment | 5 (3.3%) | 8 (5.3%) |  | 13 (4.3%) |
| Please indicate which of the following symptoms of psoriatic arthritis your patients with psoriasis or psoriatic arthritis report most often. | n | 150 | 150 |  | 300 |
|  | Joint pain, soreness, or tenderness | 114 (76.0%) | 126 (84.0%) | 0.064 | 240 (80.0%) |
|  | Swelling of fingers or toes | 9 (6.0%) | 13 (8.7%) |  | 22 (7.3%) |
|  | Fatigue | 5 (3.3%) | 2 (1.3%) |  | 7 (2.3%) |
|  | Morning stiffness | 21 (14.0%) | 8 (5.3%) |  | 29 (9.7%) |
|  | Eye problems | 1 (0.7%) | 0 |  | 1 (0.3%) |
|  | Difficulty dressing | 0 | 0 |  | 0 |
|  | Difficulty walking | 0 | 1 (0.7%) |  | 1 (0.3%) |
| Please indicate which of the following symptoms of psoriatic arthritis your patients with psoriasis and psoriatic arthritis report least often. | n | 150 | 150 |  | 300 |
|  | Joint pain, soreness, or tenderness | 4 (2.7%) | 2 (1.3%) | 0.636 | 6 (2.0%) |
|  | Swelling of fingers or toes | 4 (2.7%) | 6 (4.0%) |  | 10 (3.3%) |
|  | Fatigue | 6 (4.0%) | 9 (6.0%) |  | 15 (5.0%) |
|  | Morning stiffness | 4 (2.7%) | 4 (2.7%) |  | 8 (2.7%) |
|  | Eye problems | 108 (72.0%) | 97 (64.7%) |  | 205 (68.3%) |
|  | Difficulty dressing | 16 (10.7%) | 25 (16.7%) |  | 41 (13.7%) |
|  | Difficulty walking | 8 (5.3%) | 7 (4.7%) |  | 15 (5.0%) |
| Please indicate which of the following impacts on daily activities your patients with psoriasis or psoriatic arthritis report most often. | n | 150 | 150 |  | 300 |
|  | Difficulty with work or school activities | 25 (16.7%) | 27 (18.0%) | 0.000 | 52 (17.3%) |
|  | Difficulty with social or leisure activities | 52 (34.7%) | 16 (10.7%) |  | 68 (22.7%) |
|  | Difficulty going shopping or doing housework or yard work | 6 (4.0%) | 7 (4.7%) |  | 13 (4.3%) |
|  | Difficulty sleeping | 4 (2.7%) | 9 (6.0%) |  | 13 (4.3%) |
|  | Discomfort while doing everyday tasks | 49 (32.7%) | 88 (58.7%) |  | 137 (45.7%) |
|  | Problems with relationships | 14 (9.3%) | 3 (2.0%) |  | 17 (5.7%) |
| Please indicate which of the following impacts on daily activities your patients with psoriasis or psoriatic arthritis report least often. | n | 150 | 150 |  | 300 |
|  | Difficulty with work or school activities | 4 (2.7%) | 3 (2.0%) | 0.000 | 7 (2.3%) |
|  | Difficulty with social or leisure activities | 10 (6.7%) | 6 (4.0%) |  | 16 (5.3%) |
|  | Difficulty going shopping or doing housework or yard work | 17 (11.3%) | 11 (7.3%) |  | 28 (9.3%) |
|  | Difficulty sleeping | 64 (42.7%) | 39 (26.0%) |  | 103 (34.3%) |
|  | Discomfort while doing everyday tasks | 12 (8.0%) | 4 (2.7%) |  | 16 (5.3%) |
|  | Problems with relationships | 43 (28.7%) | 87 (58.0%) |  | 130 (43.3%) |

Note: Percentages do not include missing responses in the denominator. *P* values were computed using the Pearson chi-square test for categorical variables or the Student’s t-test for continuous variables, unless otherwise noted.

^a^ *P* value calculated using Fisher’s exact test.

1. Survey Items Most and Least Often Reported by Patients to Rheumatologists and Dermatologists

| Items | Dermatologists | | Rheumatologists | |
| --- | --- | --- | --- | --- |
|  | Most Often Reported by Patients | Least Often Reported by Patients | Most Often Reported by Patients | Least Often Reported by Patients |
| Skin symptoms (N = 200)  Itching skin  Redness of skin  Flaking skin  Painful skin  Nail problems  Difficulty choosing clothing  Embarrassment | 21.3%  24.7%  38.0%  4.7%  0.7%  0.0%  10.7% | 4.7%  2.7%  2.0%  18.0%  19.3%  50.0%  3.3% | 16.7%  26.7%  26.7%  9.3%  4.0%  0.7%  16.0% | 5.3%  4.0%  2.0%  16.7%  20.7%  46.0%  5.3% |
| Joint symptoms (N = 197)  Joint pain, soreness, or tenderness  Swelling of fingers or toes  Fatigue  Morning stiffness  Eye problems  Difficulty dressing  Difficulty walking | 76.0%  6.0%  3.3%  14.0%  0.7%  0.0%  0.0% | 2.7%  2.7%  4.0%  2.7%  72.0%  10.7%  5.3% | 84.0%  8.7%  1.3%  5.3%  0.0%  0.0%  0.7% | 1.3%  4.0%  6.0%  2.7%  64.7%  16.7%  4.7% |
| **Daily activities (N = 200)**  Difficulty with work or school  Difficulty with social or leisure activities  Difficulty shopping or doing house or yard work  Difficulty sleeping  Discomfort doing everyday tasks  Problems with relationships | 16.7%  34.7%  4.0%  2.7%  32.7%  9.3% | 2.7%  6.7%  11.3%  42.7%  8.0%  28.7% | 18.0%  10.7%  4.7%  6.0%  58.7%  2.0% | 2.0%  4.0%  7.3%  26.0%  2.7%  58.0% |
