# Supplementary material for: Comparison of US patient, rheumatologist, and dermatologist perceptions of psoriatic disease symptoms: results from the DISCONNECT study
Source: Arthritis Res Ther. 2018 May 31;20:102. doi: 10.1186/s13075-018-1601-4 (PMC5977464; doi:10.1186/s13075-018-1601-4)
Supplement: Supplementary file 5 — Relative-bother estimates for patients and physicians, grouped by survey item type. (DOCX 31 kb) [file 13075_2018_1601_MOESM5_ESM.docx]

# Additional File 5: relative-bother estimates grouped by survey item type

1. Patients’ Best-Worst Scaling Relative-Bother Weights (N = 200)

| Item | Relative-Bother Weight | 95% CI |
| --- | --- | --- |
| Skin symptoms |  |  |
| Itching skin | 0.76 | 0.64-0.87 |
| Redness of skin | 0.48 | 0.40-0.56 |
| Flaking skin | 0.62 | 0.54-0.71 |
| Painful skin | 1.03 | 0.89-1.17 |
| Nail problems | 0.33 | 0.27-0.38 |
| Difficulty choosing clothing | 0.28 | 0.23-0.32 |
| Embarrassment | 0.38 | 0.31-0.45 |
| Joint symptoms |  |  |
| Joint pain, soreness, or tenderness | 1.00 |  |
| Swelling of fingers or toes | 0.63 | 0.53-0.72 |
| Fatigue | 0.52 | 0.40-0.65 |
| Morning stiffness | 0.54 | 0.46-0.62 |
| Eye problems | 0.73 | 0.63-0.84 |
| Difficulty dressing | 0.37 | 0.31-0.42 |
| Difficulty walking | 0.86 | 0.73-0.99 |
| Impact on daily activities |  |  |
| Difficulty with work or school activities | 0.53 | 0.45-0.61 |
| Difficulty with social or leisure activities | 0.49 | 0.41-0.56 |
| Difficulty going shopping or doing housework or yard work | 0.60 | 0.51-0.70 |
| Difficulty sleeping | 0.68 | 0.58-0.78 |
| Discomfort while doing everyday tasks | 0.84 | 0.72-0.95 |
| Problems with relationships | 0.42 | 0.35-0.48 |

CI = confidence interval.

1. Physicians’ Best-Worst Scaling Relative-Bother Weights (N = 300)

| Item | Dermatologists | | Rheumatologists | |
| --- | --- | --- | --- | --- |
|  | Relative-Bother Weight | 95% CI | Relative-Bother Weight | 95% CI |
| Skin symptoms |  |  |  |  |
| Itching skin | 0.32 | 0.28-0.37 | 0.19 | 0.16-0.22 |
| Redness of skin | 0.20 | 0.16-0.24 | 0.12 | 0.09-0.14 |
| Flaking skin | 0.32 | 0.25-0.39 | 0.17 | 0.14-0.20 |
| Painful skin | 0.22 | 0.18-0.26 | 0.17 | 0.14-0.21 |
| Nail problems | 0.07 | 0.06-0.08 | 0.06 | 0.05-0.07 |
| Difficulty choosing clothing | 0.06 | 0.05-0.07 | 0.04 | 0.03-0.04 |
| Embarrassment | 0.48 | 0.41-0.54 | 0.15 | 0.13-0.18 |
| Joint symptoms |  |  |  |  |
| Joint pain, soreness, or tenderness | 1.00 |  | 1.00 |  |
| Swelling of fingers or toes | 0.44 | 0.38-0.49 | 0.57 | 0.50-0.64 |
| Fatigue | 0.16 | 0.14-0.19 | 0.22 | 0.18-0.25 |
| Morning stiffness | 0.29 | 0.25-0.33 | 0.27 | 0.23-0.31 |
| Eye problems | 0.04 | 0.03-0.05 | 0.05 | 0.04-0.06 |
| Difficulty dressing | 0.13 | 0.11-0.15 | 0.13 | 0.11-0.15 |
| Difficulty walking | 0.43 | 0.37-0.49 | 0.37 | 0.32-0.42 |
| Impact on daily activities |  |  |  |  |
| Difficulty with work or school activities | 0.49 | 0.43-0.55 | 0.54 | 0.48-0.59 |
| Difficulty with social or leisure activities | 0.48 | 0.42-0.54 | 0.27 | 0.22-0.31 |
| Difficulty going shopping or doing housework or yard work | 0.29 | 0.25-0.33 | 0.46 | 0.41-0.51 |
| Difficulty sleeping | 0.15 | 0.13-0.18 | 0.12 | 0.10-0.14 |
| Discomfort while doing everyday tasks | 0.70 | 0.63-0.77 | 0.74 | 0.68-0.80 |
| Problems with relationships | 0.20 | 0.15-0.26 | 0.13 | 0.09-0.17 |

CI = confidence interval.
